# A scoping review to identify and map the multidimensional domains of pain in adults with advanced liver disease

**DOI:** 10.1080/24740527.2020.1785855

**Published:** 2020-09-15

**Authors:** Franklin F. Gorospe, Laura Istanboulian, Martine Puts, David Wong, Elizabeth Lee, Craig M. Dale

**Affiliations:** a Lawrence S. Bloomberg Faculty of Nursing, University of Toronto, Toronto, Ontario, Canada; b Perioperative Services, Toronto General Hospital–University Health Network, Toronto, Ontario, Canada; c Acute Inpatient Respiratory Unit, Michael Garron Hospital, Toronto East Health Network, Toronto, Ontario, Canada; d Hepatology Division, Toronto General Hospital–University Health Network, Toronto, Ontario, Canada; e Department of Critical Care, Sunnybrook Health Sciences Centre, Toronto, Ontario, Canada

**Keywords:** pain, advanced liver disease, liver disease, multidimensional, biopsychosocial, scoping review

## Abstract

**Background**: Pain is a significant problem in adults living with advanced liver disease, having limited guidance available for its clinical management. While pain is considered a multidimensional experience, there have been limited reviews of the pain literature in advanced liver disease conducted with a multidimensional framework.

**Aims:** The goal of this scoping review was to identify and map the multidimensional domains of pain in adults with advanced liver disease using the biopsychosocial model.

**Methods:** We used Arksey and O’Malley’s scoping framework. A search was conducted in MEDLINE, Embase, AMED, and CINAHL databases and the gray literature using specific eligibility criteria (1990–2019). Citation selection and data extraction were performed by two independent reviewers and in duplicate.

**Results:** Of the 43 studies that met inclusion criteria, 51% were from North America and 93% utilized quantitative methods. The combined studies reported on 168,110 participants with ages ranging between 23 to 87 years. Only 9% reported an objective scoring system for liver disease severity. Few studies reported pain classification (9%) and intensity (16%). Pain prevalence ranged between 18% and 100%, with pain locations including joint, abdomen, back, head/neck, and upper/lower extremities. We identified and mapped 115 pain factors to the biopsychosocial model: physical (81%), psychological (65%), and sociocultural (5%). Only 9% measured pain using validated multidimensional tools. Pharmacological intervention (92%) prevailed among pain treatments.

**Conclusions:** Pain is not well understood in patients with advanced liver disease, having limited multidimensional pain assessment and treatment approaches. There is a need to systematically examine the multidimensional nature of pain in this population.

## RÉSUMÉ

**Contexte**: Bien que la douleur soit un problème important chez les adultes atteints d’une maladie hépatique avancée, les orientations disponibles pour sa prise en charge clinique sont limitées. Alors que la douleur est considérée comme une expérience multidimensionnelle, peu de revues de la littérature sur la douleur dans les maladies hépatiques avancées ont été menées à l’aide d’un cadre multidimensionnel.

**Objectifs:** L’objectif de cette revue exploratoire était de déterminer et de cartographier les aspects multidimensionnels de la douleur chez les adultes atteints d’une maladie hépatique avancée en utilisant le modèle biopsychosocial.

**Méthodes:** Nous avons utilisé le cadre d’Arksey et O’Malley. Une recherche a été effectuée dans les bases de données MEDLINE, Embase, AMED et CINAHL et dans la littérature grise, en utilisant des critères d’admissibilité précis (1990–2019). La sélection des citations et l’extraction des données ont été effectuées en double par deux examinateurs indépendants.

**Résultats:** Sur les 43 études répondant aux critères d’inclusion, 51 % provenaient d’Amérique du Nord et 93 % avaient utilisé des méthodes quantitatives. Les études combinées ont porté sur 168 110 participants dont l’âge variait entre 23 et 87 ans. Seuls 9 % des études ont affirmé avoir eu recours à un système de notation objective pour la gravité des maladies du foie. Peu d’études ont déclaré avoir classé la douleur (9 %) et déterminé son intensité (16 %). La prévalence de la douleur variait entre 18 et 100 % et comprenait des zones douloureuses incluant les articulations, l’abdomen, le dos, la tête et le cou, ainsi que les extrémités supérieures et inférieures du corps. Nous avons déterminé et mis en correspondance 115 facteurs de la douleur pour le modèle biopsychosocial : physiques (81 %), psychologiques (65 %) et socioculturels (5 %). Seuls 9 % mesuraient la douleur en utilisant des outils multidimensionnels validés. L’intervention pharmacoloègique (92 %) dominait parmi les traitements de la douleur.

**Conclusion:** La douleur chez les patients atteints d’une maladie hépatique avancée n’est pas bien comprise et les approches d’évaluation et de traitement multidimensionnelles de la douleur sont limitées. Il est nécessaire d’examiner systématiquement la nature multidimensionnelle de la douleur dans cette population.

## Introduction

Pain is a significant problem in adult patients living with advanced liver disease.^1–4^ Advanced liver disease is characterized by the inability of the liver to meet the metabolic needs of the body, resulting in systemic complications and eventually death.^2–4^ As a chronic and progressive illness, advanced liver disease involves cirrhosis (i.e., scarring) of the liver. Those advancing to decompensated cirrhosis and liver failure experience a number of complications, including ascites, encephalopathy, and varices. Physical pain can result from fluid retention, contributing to abdominal, joint, back, and diffuse pain. Other common sources of pain include muscle cramps, headaches, and pruritus. Recent systematic and scoping reviews indicate that as many as 79% to 82% of patients with advanced liver disease report pain.^1,5^ Common concomitant psychological symptoms of anxiety, depression, and fatigue in this population are known to amplify pain.^6–8^ Unrelieved physical and psychological symptoms may lead to persistent pain, which negatively impacts overall physical and social function in this patient population.^8–10^

No evidence-based guidelines currently exist for holistic pain management in patients with advanced liver disease.^11,12^ This is an important gap given that international reports project a substantial increase in the number of patients living with decompensated cirrhosis.^13^ Commonly used over-the-counter and prescription pain relievers such as acetaminophen, nonsteroidal anti-inflammatory drugs, and opiates are metabolized through the liver. Alterations in analgesic pharmacokinetics and metabolism during liver disease can lead to an increased risk for hepatotoxicity and accumulation of toxic metabolites.^2^ Coexistent renal disorders in this population further exacerbate drug excretion and risk for toxicity.^3^ Though patients may take lower doses of some commonly available analgesics, they may be reluctant to do so for fear of side effects.^4^ In addition, a comprehensive appraisal of pain may be overlooked during clinic visits with the health care provider due to a myriad of competing medical priorites.^2,3^ Taken together, these patients may be vulnerable to unmanaged pain, which contributes to impaired sleep, psychological distress, pain-related disability, and reduced health-related quality of life.^1,3,6^

Pain research suggests that pain is more than just a product of sensory inputs.^14–16^ Melzack and Katz^17^ and other researchers^18–20^ highlight pain as a multidimensional experience determined by the interrelationship of various internal and external domains. For instance, increased pain severity among patients with inflammatory bowel disease has been positively associated with depression and anxiety.^21^ Comparatively, patients with multiple sclerosis were found to have the quality of pain positively associated with fatigue.^20^ As a result, these researchers highlight the importance of considering pain factors beyond the physical domain. Pain is a dynamic process involving multiple domains that continuously influence each other.^17^

The biopsychosocial model of pain offers a multidimensional framework for understanding pain. As defined by Turk and Gatchel,^22^ the biopsychosocial model identifies pain as a unique, multidimensional experience influenced by a person’s physical, psychological, and sociocultural domains (see Table 1).^22–26^ Each domain plays a significant role in the dynamic process of the patient’s pain experience. Though described individually, these domains are continuously interacting with each other to shape the patient’s pain experience.^22,27^ The physical (bio-) domain addresses nociception, wherein a physiological event (e.g., injury) engages the nervous system to stimulate pain receptors. The neural signals are contextualized by the patient, who actively makes meaning of the event. The psychological (psycho-) domain determines the unique pain experience of the patient, including cognitive (beliefs, self-efficacy, cognition, and coping), affective (depression, anxiety, and anger), and personality factors. The sociocultural (socio-) domain refers to circumstances that can influence the patient’s perception, beliefs, and expectations of pain and involve the interaction between the patient and external influences. These include learned behavior through observation (social learning mechanism), social support (operant learning mechanism), and cultural beliefs (respondent learning mechanism). The biopsychosocial conceptual framework has previously led to the development of therapeutic and cost-effective interprofessional pain management programs.^22,28^

**Table 1.** Biopsychosocial conceptual model of pain

Domain of pain	Contributing factors of pain
Physical (bio-)^22^ addresses nociception, wherein a physiological event (e.g., injury) engages the nervous system to stimulate pain receptors	Musculoskeletal pain related to persistent systemic inflammation, structural body changes, and issues with the nervous system.^39^
	Visceral pain related to inflammation of internal organs, issues with blood supply, and mechanical obstruction, dilation, traction, or compression of internal tissues.^40^
	Headache pain related to cranial or vascular changes.^41^
	Paresthesia related to generalized body pain, cutaneous lesion, and pruritis.^42^
Psychological (psycho-)^22^ determines the unique pain experience of the patient, including cognitive (beliefs, self-efficacy, cognition, and coping), affective (depression, anxiety, and anger), and personality	Cognitive factors related to beliefs about pain, beliefs about controllability, self-efficacy, negative belief about one’s self or situation, and coping.^22^
	Affective factors related to depression, anxiety, and anger.^22^
	Personality factors related to the patterns unique to the individual.^22^
	Fatigue related to feeling of tiredness.^22^
Sociocultural (socio-)^22^ refers to circumstances that can influence the patient’s perception, beliefs, and expectations of pain and involve the interaction between the patient and external influences	Social learning mechanisms related to pain behavior expressions acquired through observations or modeling processes (e.g., learned behavior through observation).^22^
	Operant learning mechanisms related to external influences that can reinforce behavior (e.g., social support).^22^
	Respondent learning mechanisms related to learned associations of stimuli that are believed to increase pain (e.g., cultural beliefs).^22^

Considering the potential complexity of pain experienced by patients with advanced liver disease, reliance on a single approach (i.e., treatment of one pain domain) may result in limited success. The biopsychosocial model advocates for greater diversification of approaches to properly match pain treatment to the patient’s unique needs.^29^ Clarke et al.^30^ highlight the importance of involving interprofessional expertise such as anesthesiology, psychology, nursing, physiotherapy, and pharmacy to facilitate a multidimensional approach in addressing the physical, psychological, and sociocultural domains of pain. Multidimensional pain management strategies that holistically target these domains have been shown to be effective.^29,30^

To our knowledge there have been no reviews of the pain literature in advanced liver disease conducted using the biopsychosocial model of pain. A scoping review is a form of literature review with the purpose of mapping key concepts in an area of research that is underexplored.^31–34^ Unlike systematic reviews or meta-analysis that aim to answer questions of efficacy, scoping reviews do not narrow the boundaries of the review. A scoping review is a broad systematic exploration identifying key concepts, types of evidence, and research gaps. Because pain is a multidimensional concept, a scoping review is appropriate for the generation of a diverse range of evidence concerning completed research in addition to the identification of underexplored domains for future research inquiry.

Our specific scoping review objectives were to (1) describe the prevalence and classification of pain; (2) explore common pain characteristics (i.e., intensity, quality, and location); (3) identify physical, psychological, and sociocultural domains of pain and contributing factors (domain-specific variables that influence the patient’s experience with pain; see Table 1); (4) map recommended pain assessment and treatment strategies to the biopsychosocial framework; (5) identify gaps to aid the planning of future pain research; and (6) determine the quality of the evidence from the included studies in the advanced liver disease literature.

## Materials and Methods

We prospectively registered our search strategy with PROSPERO (CRD42019135677), and detailed review methods were based on the previously published protocol.^35^ This scoping review was informed by Arksey and O`Malley`s framework,^31^ which was advanced by Levac et al.^32^ and Colquhoun et al.^33^ The reporting of this scoping review was based on Tricco et al.’s^34^ Preferred Reporting Items for Systematic Reviews and Meta-Analyses extension for Scoping Reviews (PRISMA-ScR).

### Information Sources and Search Strategy

We conducted a comprehensive review of scholarly and gray sources of the literature that were published from January 1, 1990, to May 5, 2019, with the assistance of a health sciences information specialist and an interprofessional research team. Scholarly literature was obtained from the electronic databases Medline, Embase, AMED (Allied and Complimentary Medicine), and CINAHL (Cumulative Index to Nursing and Allied Health Literature) and focused on the major concepts of pain, symptomatic advanced liver disease, and pain assessment and management.

Development of the search terms involved consultations with the health sciences information specialist and discussions with the research team. The search strategy developed for Medline was translated to the search in Embase, AMED, and CINAHL (see Supplemental Appendix A for the Medline search strategy).

For gray sources of the literature published outside of the conventional scholarly databases,^34^ we utilized the Canadian Agency for Drugs and Technologies in Health’s^36^ guide targeting professional organizations and relevant government agencies. Professional organizations included the American Association for the Study of Liver Diseases, International Association for the Study of Pain, Canadian Liver Foundation, PBC Society of Canada, Canadian Society of Transplantation, Canadian Liver Transplant Network, Canadian Society of Intestinal Research, Canadian Association of Hepatology Nurses, Canadian Association for the Study of Liver, Canadian Liver Meeting, Canadian Network on Hepatitis C, American Liver Foundation, European Association for the Study of Liver, Canadian Association of Gastroenterology, National Pain Center, Canadian Medical Association, and the Institute for Clinical Evaluative Sciences. Government agencies included all Canadian provinces’ and territories’ websites on liver disease, public health, cirrhosis, hepatitis, and chronic disease. Other government sources were the Canadian Institute for Health Information, Statistics Canada, Health Canada, and the Public Health Agency of Canada. Additionally, Google and Google Scholar search engines were searched for the first 100 results.

### Inclusion Criteria

We used the population, concepts, and context categories specified by the Joanna Briggs Institute,^37^ which allowed for a broad scope when investigating a previously underexplored area. The population, concepts, and context categories were as follows:

- Population: Studies that included participants who were 18 years of age and older; had a primary diagnosis of advanced liver disease, advanced chronic liver disease, liver failure, end-stage liver disease, decompensated liver disease, or decompensated cirrhosis; presence of physical (e.g., joint pain, muscle cramps, skin discomfort, generalized body pain, ascites, back pain, pruritus, and headache) or psychological (e.g., anxiety, irritability, depression, and fatigue) or social (e.g., activity interference, social support) symptoms associated with pain.
- Concepts of interest: Pain prevalence, classification, characteristics, assessment, and management. Similarly, contributing factors that influence the patient’s experience with pain, as well as studies that report the assessment or management of pain.
- Context: Included studies were those from a broad sociocultural context, any geographical location, and any health care setting available in full text and published in English.
- Study design: Any type of design.

### Study Selection

Selection of the studies involved a two-stage process.^32^ The literature results were imported into EndNote X9 and advanced deduplication methods were applied.^38^ The results were then imported into Covidence, an online screening tool, where two independent screeners (F.G., L.I.) reviewed the title and abstracts for eligibility. This was followed by an independent review of the included full-text papers independently and in duplicate (F.G., L.I.). Disagreements regarding inclusion were resolved by consensus.

### Data Extraction

Comprehensive data extraction involved a two-step approach.^31,32,34^ First, two reviewers (F.G., L.I.) independently recorded the data using forms iteratively developed by the research team in Microsoft Excel. Both reviewers compared and discussed the data obtained. Consultation with a third reviewer (C.D.) was made to ensure that the data were in line with the scoping review objectives. Second, we collated the data from the included studies using tables in Microsoft Excel for analysis. Extracted data included the following:

1. Study characteristics including the name of the first author, year of publication, journal title, geographic location of the study, and study setting.
2. Methodological information of the categorization of study (quantitative, qualitative, or mixed methods), study purpose, study design, theoretical framework, data collection, and analysis methods.
3. Participant information including sample size, age, sex, categorization and diagnosis of liver disease.
4. Reported biopsychosocial factors of pain (i.e., influential variables) were extracted and mapped to three conceptual framework domains.First is the physical domain.^22^ We contextualized the physical domain to reflect the pain reports identified with advanced liver disease using the International Association for the Study of Pain definitions including musculoskeletal,^39^ visceral,^40^ headache,^41^ and paresthetic sources of pain.^42^ The second is the psychological domain that involves cognitive, affective, personality, and fatigue.^22^ The third is the sociocultural domain that considers learned behavior through observation, social support, and cultural beliefs.^22^
5. Information about pain assessment methods to determine whether pain was measured directly or indirectly as a component of another measurement tool (e.g., health-related quality of life).
6. Information about pain management interventions targeting physical, psychological, or sociocultural domains.

### Quality Appraisal

We used the Mixed Methods Appraisal Tool (MMAT) version 2018 to determine the methodological quality of each included study.^43^ The benefit of using the MMAT is its ability to assess quality between quantitative, qualitative, and mixed methods without having to rely on different tools.^43^ Though we did not exclude studies based on the MMAT results, critical appraisal of the evidence enabled us to identify methodological limitations informing research recommendations.^44^

### Synthesis of Results

Using Microsoft Excel, we summarized the descriptive information on study characteristics, methodology, and participants and organized the results according to the biopsychosocial conceptual framework.^22^ We identified, counted, and mapped the contributing factors of pain in each study to the biopsychosocial domains (see Table 1). We noted the proportional distribution of physical, psychological, and sociocultural domains across the studies relative to the overall total. Furthermore, we examined the method of pain assessment to determine whether pain was measured as a direct primary outcome or indirectly as a secondary outcome. Similarly, pain management interventions were categorized as physical, psychological, or sociocultural domain.

### Stakeholder Consultation

To foster the clinical relevance of this scoping review, we shared our scoping review findings with a group of liver disease experts comprising clinicians, researchers, and administrators on February 10, 2020, for feedback on significance, implications, and contextual applicability.^31–33^ Information from the consultation informed the reporting of this scoping review and is summarized below.

## Results

### Search Results

**Figure 1.**
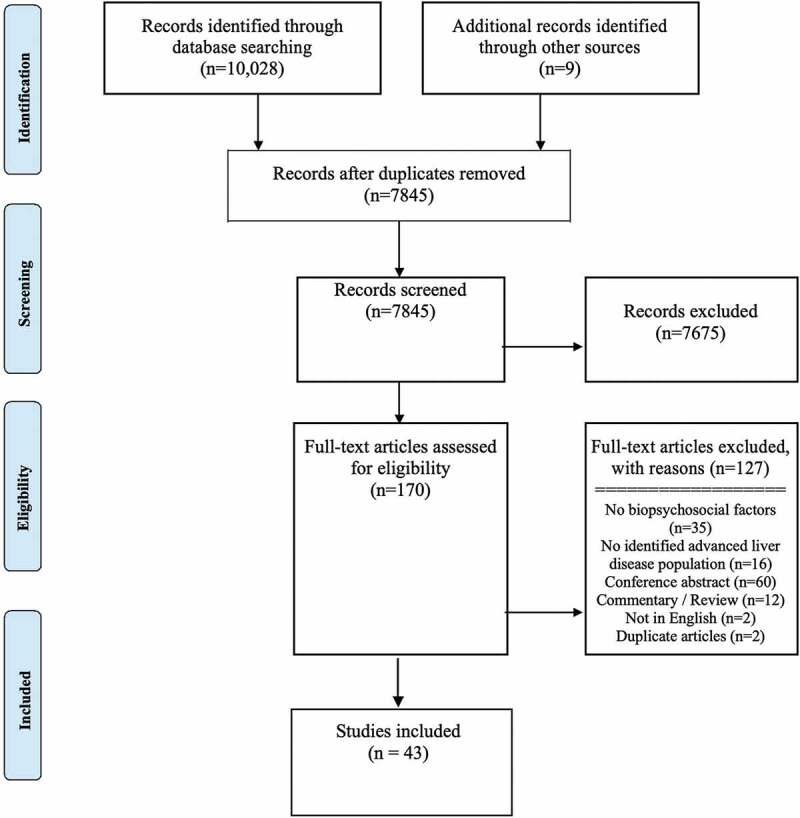
PRISMA flow diagram outlining the study selection process

Our search strategy yielded 10,037, studies including three from the gray literature (see Figure 1). After 2192 duplicates were removed, we screened 7845 articles. Based on title and abstracts we excluded 7675 articles. A total of 170 full-text articles were assessed for eligibility and 43 studies published between 1992 and 2019 met inclusion criteria.^45–87^

### Characteristics of Included Studies

A description of the included studies is provided in Supplemental Appendix B. The majority of studies were conducted in the United States (19; 44%).^50,52,54,56,59–63,70,72,75,80–86^ Other study locations included Egypt (4; 9%),^45–48^ Canada (2; 5%),^64,79^ Italy (2; 5%),^53,57^ The Netherlands (2; 5%),^68,69^ Brazil (1; 2%),^77^ Germany (1; 2%),^71^ India (1; 2%),^51^ Mexico (1; 2%),^67^ Spain (1; 2%),^78^ Turkey (1; 2%),^55^ and the United Kingdom (1; 2%).^58^ Seven (18%) studies did not report the country where the research was conducted.^49,65,66,73,74,76,77^ Study settings include inpatient (16; 37%),^45–48,50–57,59,60,79,87^ outpatient (11; 26%),^58,61,63,67,70,72,75,77,82,84,85^ and both in- and outpatient clinical settings (1; 2%).^76^ Fifteen (35%) studies did not specify the patient setting.^49,62,64–66,68,69,71,73,74,78,80,81,83,86^ A majority of the studies collected patient data in a single site (22; 51%),^46–48,53–57,59,60,67,68,70,71,75,77,79,82,84–87^ and others had two^50,52,76,81^ and three^45^ different study locations.

Of the included studies, 40 (93%) used a quantitative design (randomized controlled trials, cohort, cross section, and case series studies); 1 (2%) used qualitative (phenomenology),^49^ and 2 (5%) used mixed methods.^58,59^ Only 1 (2%) utilized a theoretical framework to guide data collection and analysis.^49^ The majority of studies (36; 84%) reported clear patient inclusion criteria, and all studies specified data collection and analysis procedures.

The number of study participants in each study varied ranging from 10^49^ to 127,239^80^ participants, with a combined total of 168,110. There was a wide range of male (29%^67^–98%^83^) and female (2%^83^–71%^67^) study participants. However, the majority of studies (34; 79%)^45–54,56,57,59,60,62,63,65,66,68,70–77,81–87^ had higher proportions of male than female participants. The age among the participants in the studies ranged from 23^68^ to 87^57^ years. Studies used a variety of diagnostic terminology or staging when reporting liver disease. For example, 1 (2%) categorized participant diagnosis as advanced liver disease.^59^ Other categorizations included unspecified cirrhosis (19; 44%),^46–51,53,54,61,62,66,70,75,77–79,83–85^ disease severity classified by the Child-Pugh method (14; 33%),^46,49,51–53,55,57,59–62,66,67,75^ model for end-stage liver disease (13; 30%),^50,54,56,70,73,75,78–81,83–85^ decompensation (2; 5%),^69,81^ and end-stage liver disease (1; 2%).^80^ Liver disease etiology varied among the studies from viral, chemical, genetic, and idiopathic injury, with 6 (14%) not specifying the primary condition.

Comorbid conditions of substance use disorders, mental health conditions, and physiological issues were identified in 35 (81%) of the studies. Substance use disorders were reported in 23 (54%) of the studies: alcohol (23; 54%), cigarettes/nicotine (5; 12%), illicit drug use (3; 7%), drug abuse (3; 7%), and heroin/narcotics (1; 2%). Mental health conditions were reported in 23 (54%) of the studies: depression (19; 44%), anxiety (16; 37%), mood disorders (2; 5%), emotional distress (1; 2%), and posttraumatic stress disorder (1; 2%). Physiological comorbid issues were reported in 14 (33%) of the studies: hepatic encephalopathy (9; 21%), diabetes (6; 14%), hypertension (4; 9%), cardiovascular disorders (3; 7%), respiratory conditions (2; 5%), hyperlipidemia (1; 2%), peptic ulcer (1; 2%), and gastrointestinal bleeding (1; 2%).

### Prevalence and Classification of Pain

The majority of studies (33; 77%) did not report pain prevalence.^45,49–69,71–74,76–78,80,81,83,85^ When reported, pain prevalence ranged from 18% to 100% (see Supplemental Appendix C).^46–48,70,75,79,82,84,86,87^ Few studies (4; 9%)^70,75,82,84^ focused on pain as a primary outcome using precise pain classification terminology such as acute, chronic, or neuropathic. For example, Hansen et al.,^70^ Madan et al.,^75^ and Rogal et al.^82,84^ utilized the term chronic pain in their study findings and reported pain according to specific body area and timing. Madan et al.^75^ reported that greater than 33% (14) of participants experienced pain in two or more bodily locations. A significant (24; 56%)^45,52,54,56–69,71,72,74,76–78,81^ number of studies reported pain as secondary outcome, meaning that pain comprised a domain within disease burden and health-related quality of life measures, and did not report pain classification.

### Common Pain Characteristics

There were minimal characteristics reported on the sensory domain of pain. Pain intensity was reported in 7 (16%) of the studies and ranged between 2 and 9 on a 0 to 10 self-report numeric scale.^46–48,70,75,82,84^ The primary location of pain was reported in 4 (9%) of the studies: joints,^69,84^ abdomen,^75,84,85^ back,^75,84^ head/neck,^75^ and upper/lower extremities.^75^ Only 1 (2%) reported qualitative descriptions of pain (e.g., aching, stabbing, sharp, and penetrating).^70^

### Biopsychosocial Factors Associated with Pain

Based on our extraction results, we identified a total of 115 contributing factors of pain according to the domains in the biopsychosocial conceptual model (see Table 1). The mapping results indicate 51% (59/115) physical, 47% (54/115) psychological, and 2% (2/115) sociocultural contributing factors of pain. The physical domain was identified in 35 (81%) studies. Common physical domain contributing factors of pain included visceral (35/59; 59%), paresthesia (14/59; 24%), musculoskeletal (8/59; 13%), and headache (2/59; 4%) pain. The psychological domain was found in 28 (65%) studies. Common psychological domain contributing factors of pain included 69% (37/54) affective (e.g., depression, anxiety), 24% (13/54) fatigue, and 7% (4/54) cognitive (e.g., beliefs about self-efficacy, pain, and controllability). The sociocultural domain of pain was found in 2 (5%) of the studies. Social support was the only identified sociocultural domain contributing factor of pain (Supplemental Appendix C). Physical–psychological domains were reported in 20 (46%) studies, and psychological–sociocultural domains were reported in 2 (5%) studies.

### Mapping the Recommendations

#### Pain Assessment Approaches

The included studies reported a limited approach to direct pain assessment. Overall, 23% (10) of studies used a direct pain measurement tool.^46–48,70,75,82,84–87^ The majority of these studies (6; 14%)^46–48,82,86,87^ used a unidimensional approach (i.e., numeric pain rating scale) primarily focused on the physical domain of pain and 4 (9%) reported use of a validated multidimensional pain assessment tool, namely, the Brief Pain Inventory^70,75^ and the McGill Pain Questionnaire.^84,85^

#### Pain Treatment Strategies

Overall, 28% (12) of the studies reported interventions to address pain.^46–48,51,70,75,80,82–86^ Pharmacological interventions to address pain were reported in 11 (92%) of these studies.^46–48,51,70,75,80,82,83,85,86^ These include medication to treat cramps (e.g., baclofen, methocarbamol, and orphenadrine),^46–48^ ascites (e.g., aldactone, furosemide and dextran),^51^ and analgesics (e.g., opioids).^70,75^ Only 4 (9%) evaluated the effectiveness of pharmacological interventions on cramps and ascites.^46–48,51^ Moreover, only one study addressed the psychological domain of pain through formal counseling intervention.^70^ None of the studies provided information on interventions that address the sociocultural domain of pain.

#### Clinical Practice Recommendations

The included studies outlined several clinical practice recommendations (Supplemental Appendix C). The most frequently reported recommendations were routine and detailed assessments of physical, cognitive, and emotional functioning (6; 14%)^54,64,70,71,76,86^; multimodal pain management strategies (6; 14%)^50,70,75,85,87^; and judicious use of pharmacological agents to treat the physical domain of pain (e.g., cramps, ascites; 6; 14%).^46–48,51,53,57^ The next commonly cited recommendations included psychological interventions (e.g., counseling; 4; 9%)^66,68,70,78^; reduced reliance on pharmacological agents (3; 7%)^76,83,86^; and targeted educational, emotional, and social support (3; 7%).^49,59,62^ Other recommendations included early consultation with palliative care^56,79^ and greater efforts to evaluate patient pain knowledge and beliefs when designing interventions.^77^ Though the included studies provided recommendations, there was a lack of evidence concerning the safety and efficacy of the suggested strategies.

### Quality Appraisal of Included Studies

The MMAT results suggests that the majority of included studies have methodological and reporting issues that contribute to bias (Supplemental Appendices D and E).^43^

Quantitative studies included randomized controlled trials (3/39; 8%), nonrandomized (35/39; 89%), and descriptive (1/39; 3%) design. Randomized controlled trials of pharmacological interventions addressed cramps^46–48^ and were single-center studies, which can limit external validity, required to support widespread changes in practice. Furthermore, lack of clarity in reporting patient characteristics such as severity of liver disease^46,49–57,59–62,66,67,70,73,75,76,78–81,83–85^/staging^45^ of cirrhosis promotes concerns with the comparability of study participant groups and whether samples are representative of real-world populations. Nonrandomized studies include cohort (11/35; 31%) and cross-sectional (24/35; 69%) designs. Some concerns for bias for nonrandomized studies include the lack of representativeness of the sample (31/35; 89%; i.e., single study site), inability to address confounders (15/35; 42%; i.e., nonresponse bias), and issues with the delivery of the intervention as intended (2/35; 6%; i.e., absence of discussion addressing intervention fidelity). Quality appraisal of the descriptive study^70^ indicates a lack of representativeness of the sample (i.e., low sample size).

The mixed methods studies have several quality issues.^58,59^ The participants comprised convenience samples drawn from single-site clinics. This introduces concern with the representativeness of the participants. As a result, the participants may not have been comparable to patients in other settings or jurisdictions. The studies did not address nonresponse bias as a potential for missing outcome data. Concerns for bias include the lack of rationale to justify the utility of a mixed methods design. There was a lack of detailed reporting in the methods for analysis between qualitative and quantitative data.^58^

Quality appraisal of the qualitative study suggests issues with the methodological design and interpretation of the results.^49^ With the absence of a research question, it is difficult to determine the appropriateness of the design and data collection method. The small sample size and limited reporting of patient characteristics pose a concern for representativeness of the sample. Furthermore, there was minimal sharing of participant response quotes to support the results and conclusions.

### Stakeholder Consultation Results

A total of 23 stakeholders from patient care (i.e., hepatologists, nurse practitioners, oncologists, pathologists, radiologists, registered nurses, and surgeons), research (i.e., investigators, research fellows), and administration (i.e., management, coordinators) attended a consultation meeting that included a presentation of our scoping review findings. Because patients with advanced liver disease frequently seek professional help due to discomforting disease symptoms, clinicians endorsed the importance of pain appraisal and management. However, stakeholders disclosed limited appraisal and reporting of pain in clinical practice, which may result in significant biopsychosocial problems. Finally, stakeholders suggested that new research is warranted to better understand patient priority pain needs, beliefs, and self-management capacities along the disease continuum.

## Discussion

The aim of this scoping review was to identify key concepts, types of evidence, and research gaps in the pain literature for patients with advanced liver disease and map our results to the biopsychosocial model of pain comprising physical, psychosocial, and sociocultural domains.

Our review confirms that pain is a substantial problem in advanced liver disease, with reported pain prevalence ranging from 18% to 100%.^46–48,70,75,79,82,84,86,87^ Issues with study design and recruitment strategies may explain the variation in pain prevalence from the included studies. The severity of liver disease determines the systemic complications and related symptoms experienced by patients.^2–4^ Considering the lack of consistently reported severity of liver disease/staging of cirrhosis in the included studies, it is unclear whether the sampling strategies account for possible differences within and across patient groups with respect to pain. Furthermore, the context of each study differs with respect to country, health care setting, and participant characteristics. These differences allow for variability in the way participants report pain and the methods clinicians use to assess pain. Therefore, the wide prevalence of pain may be a result of sampling differences.^88^

Only one third of the included studies investigated pain as a primary outcome, suggesting that the topic is not well examined and additional research is warranted. Patients report the importance of pain. Moreover, pain is often experienced in two or more body sites concurrently. However, pain classification (i.e., acute, chronic, or neuropathic) and its qualitative characteristics are not well delineated in the literature. Few studies report using multidimensional tools, rendering pain appraisal primarily unidimensional (i.e., pain intensity). Of the studies reporting pain management strategies, most were pharmacologically based interventions; few psychosocial and sociocultural pain interventions were identified.

### Implications for Pain Care

Our review of the literature demonstrates limited understanding of a relationship between physiological, psychological, and social domains in the pain experience of individuals with advanced liver disease.^1,5,12^ Contemporary pain science suggests that each of these domains cannot be considered in isolation; perturbations in one may worsen the clinical presentation of pain.^17,18,22^ Complex comorbid conditions among patients with advanced liver disease, including physiological issues, mental health conditions, and substance use disorders, reinforce the opportunity to advance pain management using a multidimensional model and an interprofessional approach.^29,30^ In the absence of studies reporting multifaceted interventions based upon a multidimensional model, patients with advanced liver disease may experience suboptimal pain management.

Though most studies focused on the physical pain domain and pharmacological treatment, we found limited drug safety and efficacy research in our review. We retrieved only three randomized controlled trials informing pharmacological pain treatment.^46–48^ Studies emphasize the challenge of balancing the benefits and risks of analgesic dosing with potential systemic complications, including encephalopathy, renal injury, and bleeding.^8,89,90^ In the absence of studies examining the safety and efficacy of pharmacological treatments, evidence informing pain management for this population remains limited.^91^

Frequent mention of the psychological pain domain may be due to the fact that some patients with advanced liver disease present with mental health comorbidity and/or substance use disorders.^92,93^ Cognitive and emotional factors have an important influence on pain experience. Moreover, these conditions can impede the patient’s ability to interact with caregivers, thereby leading to poor pain management.^94^ We found affective (e.g., depression, anxiety, and fatigue)^95^ and cognitive (e.g., beliefs)^96,97^ pain factors to predominate in our review. However, only one treatment comprising counseling was noted.^68^ Counseling has been shown to improve pain in other patient populations.^95,98^ Talk therapy may allow patients to discuss their psychological state, disclose priority concerns, and develop strategies to address them.

Social circumstances and culturally specific attitudes and beliefs about pain are known to influence the manner in which individuals view and respond to pain.^17,18,22,95^ Socioeconomic factors (e.g., lower levels of education and income) correlate with higher pain perception and incidence of chronic pain.^27^ Cultural factors related to the pain experience include pain expression, pain language, and expectations for support.^17,22^ However, we found few studies reporting the sociocultural domain of pain.^49,59^ One possible explanation for the low reporting of the sociocultural domain is limited integration of the health locus of control concept in pain care.^99–101^ In this framework, individuals believe that they are either in control or not in control of their health.^87^ Patients experiencing physical and mental helplessness may demonstrate social disengagement, characterized by the absence of a social network.^88,91^ Importantly, patients may not understand how the sociocultural domain (i.e., loneliness) influences pain.^102^ Lack of social support has been clearly associated with poor pain outcomes in other populations.^103–105^ Possible strategies to facilitate social connections include counseling, social support groups, and palliative care consultation.^59^ Interventions aimed at decreasing loneliness may simultaneously reduce pain.^106^

### Implications for Research

A key research gap is pain measurement and reporting in the literature involving patients with advanced liver disease. We found insufficient use of multidimensional pain assessment tools.^70,75,84,85^ Lack of studies exploring pain as a multidimensional concept may contribute to an insufficient understanding of the relationship between physical, psychological, and sociocultural domains. Other gaps include reporting of the characteristics, typology, and implications of pain in this population. Researchers should investigate multidimensionality of pain to identify and better understand potentially modifiable patient needs.

Considering the lack of consistency in diagnostic terminology when categorizing (i.e., stage or classification) patients with advanced liver disease, synthesizing research evidence may result in inconsistencies. Our review findings suggests that the study samples vary significantly, making it difficult to conduct systematic reviews to inform pain interventions.^46–48,70,75,79,82,84,86,87^ Durand and Valla^107^ and Peng et al.^108^ provide key discussions on the utility of advanced liver disease severity scoring as a way to classify patients. For example, the Child-Pugh and model for end-stage liver disease (MELD) score models have been shown to offer a consistent method for classifying liver disease severity.^107,108^ We recommend that future research report precise classification of advanced liver disease and physical/psychological comorbidities.

Given the prevalence of pain among patients with advanced liver disease, there is a need to evaluate the effectiveness of pharmacological and nonpharmacological interventions.^95,98,109^ Investigation of the effectiveness and associated risks of opioid and nonopioid analgesic medications is required. Similarly, evaluation of the impact of behavioral strategies, alone or in combination with pharmacological strategies (i.e., multimodal treatment), may be explored. Unidimensional pain intensity tools are important for providing control and intervention measures of pain intensity in response to investigative treatment. Moreover, studies employing multidimensional pain tools such as the McGill Pain Questionnaire and Brief Pain Inventory will allow further insight into the impact of pain treatment on function and/or quality of life^43,110,111^ and enhance understanding of pain characteristics and classification.

Patient-oriented research investigating patient pain experiences, priorities, and self-management strategies may increase the relevance of research investment in this domain. Future exploratory research may include patient/family pain beliefs, pain interference on activities of daily living, pain self-management strategies, help-seeking experiences, and socioeconomic factors that may influence the pain experience. In support of this aim, greater use of qualitative and mixed methods studies targeting the patient/family experience of pain and its clinical management may be of assistance. Inclusion of vulnerable patient groups including those with mental health and substance use disorders requires special consideration. Hansen et al.^70^ suggest that patient pain self-management strategies can potentially reveal effective treatment approaches (e.g., rest, support, and relaxation techniques). Exploring self-care pain management strategies can help researchers develop targeted multidimensional interventions.

## Strengths and Limitations

Our review had some limitations. First, although we utilized a comprehensive and systematic scoping strategy, our scholarly information sources were limited to four databases. Similarly, our gray literature search was limited to professional organizations, government agencies, and internet search methods. The search may have missed relevant articles due to the lack of indexing terminology specific to substance use disorders and mental health diagnoses, which may be common in some liver disease patient populations. Second, we only included articles published in English. Countries with a growing patient population diagnosed with liver disease (e.g., China, France, Japan)^12^ may offer relevant non-English-language studies that can expand our findings. Third, most of the studies are from economically developed countries, which limited our ability to explore pain research in other countries. Fourth, our stakeholder consultation meeting did not include patients, family caregivers, or pain clinicians, which may limit our interpretation and recommendations. Finally, by electing to conduct a scoping review that is broad and inclusive of what is known in the literature, we sacrifice specific details that may be important to clinicians for patient care. Our review also had strengths, including the use of a multidimensional pain model to map the results. Attention to physical, psychological, and sociocultural domains of pain enabled a view to potentially modifiable factors influencing pain management in advanced liver disease. Additional strengths include the use of a search strategy informed by a health information expert, PRISMA-ScR reporting structure, and dual reviewer extraction and coding.

## Conclusion

In our scoping review of patients with advanced liver disease, we found that there is a lack of research focused on pain as a primary outcome using precise pain classification. Based on the limited studies available, pain was highly prevalent and frequently assessed as a unidimensional physical phenomenon managed primarily through pharmacological strategies. Our results demonstrate limited qualitative and mixed methods research investigating the patient experience of pain and its clinical management. Future research should investigate the use of multidimensional pain appraisal tools and explore the patient’s experience with pain to better inform the development of effective multidimensional pain management strategies for this growing population.

## Supplementary Material

Supplemental Material

Supplemental Material

Supplemental Material

Supplemental Material

Supplemental Material

## Data Availability

All available data in this scoping review are referenced throughout the article as Figure 1; Table 1; Supplemental Appendices A, B, C, D, and E.

## Author Details

The research team made significant intellectual contributions to the development of this scoping review. FG and CD conceptualized the methodological design of this review and provided guidance to the research team. FG, CD, MP, DW, and EL were involved in development of the scoping review questions and design. FG and LI initially developed the data extraction framework, which was then further developed by input from the research team. FG and CD initiated the first draft of the article, which was then followed with substantial input from all of the authors. All authors approved the final version of the article.

## Data Deposition

No other data.
