# Supplementary material for: A scoping review to identify and map the multidimensional domains of pain in adults with advanced liver disease
Source: Can J Pain. 2020 Sep 15;4(1):210–24. doi: 10.1080/24740527.2020.1785855 (PMC7951148; doi:10.1080/24740527.2020.1785855)
Supplement: Supplemental Material [file UCJP_A_1785855_SM7879.docx]

Supplemental Appendix A. Medline search strategy

| **#** | **Searches** | **Results** |
| --- | --- | --- |
| 1 | pain.tw,kf. | 571308 |
| 2 | ((abdominal or acute or arthralgi* or joint or back or muscle or musculoskeletal or general* body or chronic or flank or headache or nociceptive or intractable or postoperative or procedural or referred or persistent or constant or consistent or visceral or somatic or psychosomatic or regional) adj1 pain).tw,kf. | 180786 |
| 3 | (anxiety or irritability or irritable or depression or depressed or fatigue or tired or tiredness or anger).tw,kf. | 569346 |
| 4 | (ascites or cramp*).tw,kf. | 47217 |
| 5 | ((hepatic or liver) adj1 encephalopathy).tw,kf. | 7401 |
| 6 | (quality of life or symptom).tw,kf. | 415955 |
| 7 | or/1-6 [Concept 1 = Pain] | 1440503 |
| 8 | end stage liver disease.tw,kf. | 8164 |
| 9 | (diseas* adj1 liver).tw,kf. | 96918 |
| 10 | ((liver or hepatic) adj1 failure*).tw,kf. | 25175 |
| 11 | (dysfunction* adj1 liver).tw,kf. | 7464 |
| 12 | cirrhos*.tw,kf. | 89286 |
| 13 | ((decompensate* or liver or hepatic) adj1 cirrhos*).tw,kf. | 37338 |
| 14 | ((liver or hepatic or failure*) adj1 decompensate*).tw,kf. | 1149 |
| 15 | (fibros* adj1 liver).tw,kf. | 14313 |
| 16 | or/8-15 [Concept 2 = Liver Disease] | 191849 |
| 17 | ((management* or intervention*) adj1 pain).tw,kf. | 22632 |
| 18 | ((non-pharmacological or nonpharmacological) adj2 pain).tw,kf. | 234 |
| 19 | (transcutaneous electrical nerve stimulation or tens or acupuncture or electroacupuncture or acupressure or warming or heat).tw,kf. | 250641 |
| 20 | (pharmacolog* adj2 pain).tw,kf. | 794 |
| 21 | (acetaminophen or nsaid* or nonsteroidal anti-inflammatory drug* or nonsteroidal antiinflammatory drug* or antidepressant* or anticonvulsant* or anesthetic* or anaesthetic* or opioid* or snri or serotonin-norepinephrine reuptake inhibitor* or serotonin norepinephrine reuptake inhibitor* or tramadol or hydrocodone or oxycodone or morphine or hydromorphone or oxycodone or morphine or hydrocodone or methadone or codeine or fentanyl or meperidine or marijuana or analgesic*).tw,kf. | 400226 |
| 22 | paracentesis.tw,kf. | 3072 |
| 23 | ((measurement* or scale* or assessment* or questionnaire* or test* or analog or visual or visual analog*) adj1 pain).tw,kf. | 20830 |
| 24 | (health related quality of life or hrqol or quality of life or qol).tw,kf. | 252338 |
| 25 | or/17-24 [Concept 3 = Pain Assessment & Management] | 914256 |
