# Supplementary material for: A scoping review to identify and map the multidimensional domains of pain in adults with advanced liver disease
Source: Can J Pain. 2020 Sep 15;4(1):210–24. doi: 10.1080/24740527.2020.1785855 (PMC7951148; doi:10.1080/24740527.2020.1785855)
Supplement: Supplemental Material [file UCJP_A_1785855_SM7873.docx]

Supplemental Appendix B. Study characteristics of published studies (n = 43)

| **Quantitative design: randomized controlled trials** | | | | | | | | | |
| --- | --- | --- | --- | --- | --- | --- | --- | --- | --- |
| 1 | Abd-Elsalam S. (2018)^47^ | To evaluate the efficacy and safety of methocarbamol as a novel therapy in controlling muscle cramps in cirrhotic patients. | Egypt (inpatient). | Randomized placebo controlled double blind trial. | Liver cirrhosis; frequent muscle cramps. | 100 (55.1 +/- 8.2 years). | None reported. | None reported. | M = 65 (65%)  F = 35 (35%) |
| 2 | Abd-Elsalam S. (2017)^48^ | To assess the efficacy and safety of orphenadrine in the treatment of muscle cramps in patients with liver cirrhosis. | Egypt (inpatient). | Randomized controlled double blind parallel group pilot study. | Liver cirrhosis; frequent muscle cramps. | 30 (51.4 +/- 5.71 years). | None reported. | None reported. | M = 18 (60%)  F = 12 (40%) |
| 3 | Elfert AA. (2016)^46^ | To assess the safety and effectiveness of baclofen in the treatment of muscle cramps in patients with liver cirrhosis. | Egypt (inpatient). | Randomized placebo controlled trial. | Liver cirrhosis; frequent muscle cramps. | 100 (52.5 +/- 7.74 years) . | None reported. | None reported. | M = 52 (52%)  F = 48 (48%) |
| **Quantitative design: non-randomized studies** | | | | | | | | | |
| 4 | Acharya C. (2017)^50^ | To explore association of opioid use on hospital discharge with readmission and functional status of patients with liver cirrhosis. | USA (inpatient). | Prospective cohort design. | Liver cirrhosis; capable of informed consent. | 144 (56 +/- 9.07 years). | None reported. | Alcohol related liver disease, diabetes, hepatic encephalopathy. | M = 85 (59%)  F = 59 (41%) |
| 5 | Acharya C. (1992)^51^ | To evaluate the efficacy of a low-cost, easily available synthetic colloid-like, low molecular weight dextran infusion as the plasma expander in preventing hypovolemia, electrolyte disturbances, and renal dysfunction in patient with cirrhosis and tense ascites subjected to large volume paracentesis. | India (inpatient). | Quasi-experimental cohort study. | Liver cirrhosis; absence of encephalopathy or gastrointestinal bleeding; absence of bacterial infection, renal failure, cardiac disease, respiratory disease; specific blood values (serum bilirubin, liver function tests, complete blood count, urine sodium). | 40 (43.4 +/- 14.45 years). | None reported. | Alcohol related liver disease. | M = 30 (75%)  F = 10 (25%) |
| 6 | Afendy A. (2009)^52^ | To explore the impact of different types of liver disease and clinicodemographic factors on health related quality of life in a large cohort of patients with chronic liver disease. In addition, we aimed to develop models that can predict health related quality of life scale scores for Short-Form 36. | USA (inpatient). | Prospective cohort study. | Chronic liver disease. | 1103 (54.2 +/- 12.0 years). | None reported. | Depression, alcohol related liver disease. | M = 666 (60.4%)  F = 437 (39.6%) |
| 7 | Baumann AJ. (2015)^56^ | To determine if early palliative care intervention would reduce the symptom burden and depressive symptoms associated with end-stage liver disease. | USA (inpatient). | Longitudinal cohort multidisciplinary interventional study. | End-stage liver disease. | 50 (58.9 +/- 6.37 years). | None reported. | Depression. | M = 38 (76%)  F = 12 (24%) |
| 8 | Dan AA. (2008)^62^ | To assess the health utilities of patients with chronic liver disease using the SF-6D and HUI-2. We also compare utility scores among patients with different types of liver disease. | USA (Not reported). | Retrospective cohort descriptive study. | Hepatitis B, C, or cholestatic liver disease. | 140 (49.4 +/- 10.74 years). | None reported. | None reported. | M = 81 (58%)  F = 59 (42%) |
| 9 | Dan AA. (2006)^63^ | To examine the association of health related quality of life with treatment-induced depression and anemia in patients with chronic hepatitis C. | USA (outpatient). | Descriptive cohort survey study. | Hepatitis C who received pegylated interferon alfa 2b and ribavirin. | 271 (47.1 +/- 6.5 years). | None reported. | Depression, anxiety, alcohol related liver disease. | M = 189 (69%)  F = 82 (31%) |
| 10 | Macdonald S. (2019)^74^ | To determine the association between health related quality of life, using the SF‐36, and mortality in patients with liver cirrhosis and severe ascites. | Not reported. | Retrospective cohort study. | Liver cirrhosis; ascites managed with diuretics and therapeutic paracentesis. | 405 (Median 58 years; Range 51-66 years). | Participants with incomplete data on questionnaire and missing specific blood work data were excluded. | Alcohol related liver disease. | M = 294 (73%)  F = 111 (27%) |
| 11 | Randall HB. (2017)^80^ | To quantify fills for prescription opioids on the waiting list, identify correlates of opioid use, and determine whether prescription opioid exposure before and after liver transplant is associated with post transplant outcomes. | USA (Not reported). | Retrospective cohort study. | Liver disease; pharmaceutical fill record while on transplant waiting list. | 29637 (mean and SD not reported / 18-greater than 60 years). | None reported. | Diabetes, hypertension, cerebrovascular disease, peripheral vascular diseases, chronic obstructive pulmonary disease, coronary artery disease. | M = 19679 (66%)  F = 9958 (34%) |
| 12 | Rogal S. (2019)^83^ | To assess the longitudinal trends and predictors of opioid prescribing in a large national sample of veterans with cirrhosis. | USA (Not reported). | Retrospective cohort study. | Liver cirrhosis; opioid prescription. | 127239 (58 +/- 9 years). | Statistical models were fitted to assess for the importance of participants with and without missing data. When variables with missing values (MELD, marital status, and race) were excluded from the models, there was little change in the point estimates and relationships between the other variables. | Post-traumatic stress disorder, mood disorders, alcohol related liver disease, nicotine use disorder, | M = 124587 (98%)  F = 2652 (2%) |
| 13 | Rogal S. (2013)^82^ | To understand factors associated with pain and its treatment in patients with chronic liver disease in order to find potentially modifiable targets for intervention. | USA (outpatient). | Retrospective cohort study. | Chronic liver disease. | 1286 (52.6 +/- 12.96 years). | None reported. | Depression, anxiety, alcohol related liver disease, nicotine use disorder, illicit drug use disorder, hepatic encephalopathy. | M = 656 (51%)  F = 630 (49%) |
| 14 | Roth K. (2000)^87^ | To understand patterns of care and end-of life preferences for patients dying with end stage liver disease with cirrhosis. | Not reported (inpatient). | Prospective cohort study. | Liver cirrhosis; specific blood values (serum albumin); uncontrolled ascites; encephalopathy; cachexia; gastrointestinal bleeding. | 575 (52.6 years / SD not reported; range 42-67 years). | None reported. | Depression, anxiety, alcohol related liver disease, hepatic encephalopathy, gastrointestinal bleed. | M = 358 (62%)  F = 217 (38%) |
| 15 | Abd El-Wahab EW. (2016)^45^ | To assess the impact of chronic viral liver diseases on health related quality of life among Egyptian patients compared to an interventional group of patients undergoing interferon therapy and a control group of noninfected individuals via health related quality of life specific assessment instruments focusing on liver disease. | Egypt (inpatient). | Comparative cross sectional design. | Hepatitis C; aged 18-60; both biological sexes; specific blood values (complete blood count). | 450 (46.9 +/- 10.8 years). | None reported. | Alcohol related liver disease, smoking, drug abuse, diabetes, hypertension, hepatic encephalopathy. | M = 323 (72%)  F = 127 (28%) |
| 16 | Angeli P. (1996)^53^ | To evaluate the prevalence of cramps in cirrhosis according to cause and severity of the disease. In addition, studies were performed to define its pathophysiology. | Italy (inpatient). | Cross sectional descriptive study. | Liver cirrhosis; absence of encephalopathy, bacterial infection, gastrointestinal bleed; ascites. | 170 (57 +/- 11 years). | None reported. | None reported. | M = 109 (64%)  F = 61 (36%) |
| 17 | Barboza K. (2016)^54^ | To assess the relationship between depression, hepatic encephalopathy, and health related quality of life among cirrhotic patients with hepatitis c virus. | USA (inpatient). | Cross sectional descriptive study. | Liver cirrhosis; hepatitis C; encephalopathy. | 43 (57.8 +/- 7 years). | None reported. | Hepatic encephalopathy. | M = 27 (63%)  F = 16 (37%) |
| 18 | Baskol M. (2014)^55^ | To determine the prevalence of muscle cramps in patients with liver cirrhosis and to identify factors associated with their development, especially serum zinc. | Turkey (inpatient). | Cross sectional survey design. | Liver cirrhosis; absence of encephalopathy, bacterial infection, gastrointestinal bleed; ascites. | 185 (52.1 +/- 9.63 years). | None reported. | None reported. | M = 89 (48%)  F = 96 (52%) |
| 19 | Bianchi G. (2005)^57^ | To measure psychological well-being in patients with cirrhosis and relation of psychological status to liver function and clinical signs/symptoms. | Italy (inpatient). | Cross sectional survey design. | Liver cirrhosis; portal hypertension. | 156 (median 65 years; range 37-87 years). | None reported. | Depression, anxiety, alcohol related disease, hepatic encephalopathy. | M = 85 (55%)  F = 71 (45%) |
| 20 | Bondini S. (2007)^60^ | To assess the health related quality of life scores of patients with hepatitis B virus using validated health related quality of life instruments, and compare the health related quality of life scores of patients with hepatitis B virus versus patients with hepatitis C virus and with normative data. | USA (inpatient). | Cross sectional survey design. | Hepatitis B, C, or primary biliary liver cirrhosis. | 146 (47.2 +/- 10.6 years). | None reported. | Anxiety, chemical dependency. | M = 86 (59%)  F = 60 (41%) |
| 21 | Chatrath H. (2012)^61^ | To determine the prevalence, characteristics, and predictors of muscle cramps in patients with cirrhosis and their impact on quality of life as measured by the chronic liver disease questionnaire. | USA (outpatient). | Cross sectional survey design. | Liver cirrhosis; frequent muscle cramps. | 150 (56 +/- 10 years). | None reported. | Alcohol related liver disease. | M = 61 (41%)  F = 89 (59%) |
| 22 | Evon D. (2016)^64^ | To (a) assess fatigue using one of the PROMIS short form surveys and (b) identify clinical, laboratory, and demographic factors associated with fatigue in a large multiethnic North American population of patients with Hepatitis B. | Canada (Not reported). | Multicenter cross sectional study. | Hepatitis B; aged greater than two years. | 948 (median 42 years; range 34-52 years). | None reported. | Diabetes, hypertension, hyperlipidemia. | M = 462 (49%)  F = 478 (51%) |
| 23 | Fontana RJ. (2001)^65^ | To determine whether variability in health related quality of life scores in a cohort of patients with compensated chronic hepatitis C who had failed previous interferon therapy could be explained in part by demographic and extrahepatic clinical variables. | Not reported. | Cross sectional survey design. | Chronic hepatitis C; aged 18 years or older; specific blood values (liver function tests). | 107 (44.5 +/- 5.4 years). | None reported. | Depression, alcohol related liver disease. | M = 77 (72%)  F = 30 (28%) |
| 24 | Fritz E. (2009)^66^ | To examine the association of gastrointestinal symptoms with quality of life and psychological distress in patients with liver cirrhosis. | Not reported. | Cross sectional survey design. | Liver cirrhosis. | 75 (57 +/- 1.4 years; range 24-82 years). | None reported. | Depression, anxiety, alcohol related liver disease, diabetes. | M = 42 (56%)  F = 33 (44%) |
| 25 | Gallegos-Orozco JF. (2003)^67^ | To evaluate health related quality of life and depression in a sample of Mexican chronic hepatitis C patients naive to treatment attending a tertiary-referral care center as well as to determine patient illness understanding and knowledge of the disease. | Mexico (outpatient). | Cross sectional descriptive study. | Chronic hepatitis C. | 157 (Mean, SD, Range not reported). | None reported. | Depression. | M = 45 (29%)  F = 112 (71%) |
| 26 | Gutteling JJ. (2007)^68^ | To: (1) identify the most relevant domains of health related quality of life impairment in patients with various chronic liver diseases; and (2) assess predictors given by the liver and comorbid somatic and psychiatric diseases of health related quality of life measured by a generic and disease-specific health related quality of life instrument in patients with various chronic liver diseases. | Netherlands (Not reported). | Cross sectional survey study. | Chronic liver disease. | 32 (52.5 +/- 11.1 years; range 23-65 years). | None reported. | Depression, anxiety, alcohol related liver disease. | M = 22 (69%)  F = 10 (31%) |
| 27 | Gutteling JJ. (2006)^69^ | To assess the impact of physical and psychosocial determinants on a weighted score of health-related quality of life in patients with chronic liver disease. | Netherlands (Not reported). | Cross sectional survey study. | Chronic liver disease. | 1175 (48 +/- 12 years). | None reported. | Depression. | M = 497 (42%)  F = 678 (58%) |
| 28 | Hauser W. (2004)^71^ | To: (1) identify the most relevant domains of health related quality of life impairment in patients with various chronic liver diseases; and (2) assess predictors given by the liver and comorbid somatic and psychiatric diseases of health related quality of life measured by a generic and disease-specific health related quality of life instrument in patients with various chronic liver diseases. | Germany (Not reported). | Cross sectional survey design. | Chronic liver disease. | 203 (52.7 +/- 13.9 years). | None reported. | Depression, anxiety, alcohol related liver disease. | M = 107 (53%)  F = 96 (47%) |
| 29 | Kallman J. (2007)^72^ | To assess systematically health-related quality of life in patients with chronic hepatitis C and to determine if any clinical, biochemical, virologic, demographic, and histologic features are associated with health related quality of life status. | USA (outpatient). | Cross sectional observational study. | Chronic hepatitis C. | 130 (45 years / SD not reported). | None reported. | None reported. | M = 83 (64%)  F = 47 (36%) |
| 30 | Kaltsakas G. (2013)^73^ | To determine the prevalence of chronic dyspnea and the interrelationships among chronic dyspnea, measured with the modified Medical Research Council scale, respiratory muscle strength, and lung function in patients with end-stage liver disease. | Not reported. | Cross sectional descriptive study. | End-stage liver disease; ability to perform lung function tests; stable clinical and functional state for at least four weeks before testing. | 40 (52 +/- 10 years). | None reported. | Chronic dyspnea. | M = 29 (73%)  F = 11 (27%) |
| 31 | Madan A. (2012)^75^ | To describe the nature of chronic pain in patients with end-stage liver disease, the extent to which pain affects daily level of functioning, and the variety and effectiveness of current treatments. | USA (outpatient). | Retrospective cross sectional chart review study. | End-stage liver disease; availability of Model for End-stage Liver Disease (MELD) scores; ability to participate in psychological testing. | 108 (51.1 +/- 10.33 years). | None reported. | Alcohol related liver disease. | M = 58 (54%)  F = 42 (46%) |
| 32 | Marchesini G. (2001)^76^ | To investigate factors associated with poor perceived health status in patients with cirrhosis. | Not reported. (inpatient & outpatient). | Cross sectional survey design. | Liver cirrhosis; adult patients. | 544 (60 +/- 11 years). | In a subset of 84 cases of a single center, 13 participants had missing data. A paired analysis of final data failed to show any significant difference between mean values calculated before and after the checking of questionnaires and the interview on missing data. | Alcohol related liver disease, diabetes, peptic ulcer. | M = 348 (64%)  F = 196 (36%) |
| 33 | Paglione HB. (2019)^77^ | To evaluate the quality of life, religiosity and symptoms of anxiety and depression in liver transplantation candidates. | Brazil (outpatient). | Descriptive cross sectional study. | Liver disease; on active waiting list for liver transplantation; aged 18 years or older; both biological sexes. | 50 (52.5 +/- 12.1 years). | None reported. | Depression, anxiety, alcohol related liver disease. | M = 29 (58%)  F = 21 (42%) |
| 34 | Perez-San-Gregorio MA. (2012)^78^ | To compare the biopsychosocial functioning among liver transplantation and cirrhotic patients as a function of self-perceived pain level. | Spain (Not reported). | Cross sectional comparative design. | Liver cirrhosis; aged 18 years and older; able to cognitively complete questionnaires. | 34 (56.59 +/- 7.13 years). | None reported. | Depression, anxiety, alcohol related liver disease. | Not reported. |
| 35 | Poonja Z. (2014)^79^ | To determine: 1. Once deemed palliative (i.e., delisted or declined for liver transplant), are patients properly assessed by palliative care services, and do they have appropriate goals of care discussions? 2. What symptoms were important in the patient care, and were they alleviated? 3. How often do patients receive invasive medical interventions including renal replacement therapy and transfer to the intensive care unit? | Canada (inpatient). | Retrospective cross sectional chart review study. | Decompensated liver cirrhosis; delisted from liver transplant waiting list; aged 18 years or older. | 102 (55 +/- 9 years). | In the event of missing data values, data were not replaced or estimated. | Depression, anxiety, alcohol relate liver disease. | M = 68 (67%)  F = 34 (33%) |
| 36 | Rodrigue JR. (2010)^81^ | To: (1) characterize the severity and nature of fatigue and sleep quality before and after liver transplantation, (2) to assess the relationship between fatigue and sleep quality and quality of life, and (3) to identify their multivariate correlates. | USA (Not reported). | Cross sectional survey design. | Liver disease; on active waiting list for liver transplantation. | 110 (52.1 +/- 8.1 years). | Preliminary analyses were conducted to examine the underlying distributional properties of all outcome variables and covariates and patterns of missing data. Missing data (3% of all data points) were replaced by using the multiple imputation strategy. | Alcohol related liver disease, drug abuse, cigarette use. | M = 61 (55%)  F = 49 (45%) |
| 37 | Rogal S. (2015)^84^ | To: (1) understand the relationship between pain, inflammation, and psychiatric symptoms; (2) evaluate the factors associated with abdominal pain; (3) understand the current management of pain and predictors of prescription opioid use; and (4) assess the predictors of pain-related disability in a cohort of patients with cirrhosis. | USA (outpatient). | Prospective cross sectional survey study. | Liver cirrhosis from alcohol, hepatitis C, or nonalcoholic steatohepatitis (NASH); aged 18 years or older. | 193 (57.9 +/- 8.86 years). | None reported. | Depression, anxiety, alcohol related liver disease, tobacco, heroin / narcotics, illicit drug use, hepatic encephalopathy. | M = 116 (60%)  F = 77 (40%) |
| 38 | Rogal S. (2015)^85^ | To: (1) determine the prevalence of fibromyalgia using a validated diagnostic instrument in patients with three different etiologies for liver cirrhosis; and (2) investigate clinical correlates of fibromyalgia in the total sample. | USA (outpatient). | Cross sectional survey study. | Liver cirrhosis from alcohol, hepatitis C, or nonalcoholic steatohepatitis; aged 18 years or older. | 193 (53.6 +/- 8.88 years). | None reported. | Depression, anxiety, alcohol related liver disease, hepatic encephalopathy. | M = 116 (60%)  F = 77 (40%) |
| 39 | Rogal S. (2013)^86^ | To assess factors associated with healthcare utilization in patients with chronic liver disease with a focus on pain, opioid use, and psychiatric symptoms. | USA (Not reported). | Retrospective cross sectional study. | Chronic liver disease expected to last more than six months. | 1286 (52.6 +/- 13.03 years). | None reported. | Mood symptoms, emotional distress, alcohol related liver disease, nicotine use disorder, illicit drug use, hepatic encephalopathy. | M = 650 (51%)  F = 636 (49%) |
| **Quantitative design: Descriptive studies** | | | | | | | | | |
| 40 | Hansen L. (2014)^70^ | To provide a longitudinal description of pain characteristics in outpatients with end stage liver disease approaching the end of life, to describe variability in pain between and within patients over time, and to describe both pharmacological and nonpharmacological pain management strategies used. | USA (outpatient). | Prospective, longitudinal case series, descriptive pilot study. | Liver cirrhosis; documented patient report of pain; Model for End-stage Liver Disease (MELD) score of 18 or greater. | 20 (59 +/- 13.9 years). | None reported. | Alcohol related liver disease. | M = 15 (75%)  F = 5 (25%) |
| **Mixed Methods Design** | | | | | | | | | |
| 41 | Blackburn P. (2007)^58^ | To explore psychological factors and coping strategies in fatigued primary biliary cholangitis patients. | UK (outpatient). | Cross sectional exploratory sequential mixed method study. | Primary biliary cholangitis; specific blood values (serum liver biochemistry, serum antimitochondrial antibody, liver histology); experience of fatigue. | 24 (57.9 +/- 7.3 years). | None reported. | Depression, anxiety. | Not reported. |
| 42 | Blasiole JA. (2006)^59^ | To systematically examine the impact of the hepatitis C virus diagnosis on patients’ level of social support in a large-scale study. | USA (inpatient). | Cross sectional convergent mixed method study. | Hepatitis C; aged 18 years or older; ability to provide informed consent. | 342 (45.2 +/- 9.2 years). | Participants with missing social support data were excluded. | Depression, anxiety. | M = 214 (63%)  F = 128 (37%) |
| **Qualitative design** | | | | | | | | | |
| 43 | Abdi F. (2015)^49^ | To describe the experiences of individuals with liver cirrhosis during critical illness. The aim was to investigate the disease experiences of liver cirrhosis. | Not reported. | Colaizzi’s phenomenological qualitative study. | Liver cirrhosis; no other chronic illnesses; able to provide informed consent. | 10 (range 39-54 years). | None reported. | None reported. | M = 7 (70%)  F = 3 (30%) |
