# Supplementary material for: A scoping review to identify and map the multidimensional domains of pain in adults with advanced liver disease
Source: Can J Pain. 2020 Sep 15;4(1):210–24. doi: 10.1080/24740527.2020.1785855 (PMC7951148; doi:10.1080/24740527.2020.1785855)
Supplement: Supplemental Material [file UCJP_A_1785855_SM7871.docx]

Supplemental Appendix C. Mapping biopsychosocial factors of pain (n = 43)

| **Quantitative design: randomized controlled trials** | | | | | | | | |
| --- | --- | --- | --- | --- | --- | --- | --- | --- |
| 1 | Abd-Elsalam S. (2018)^47^ | Not reported (NR). | Numerical Rating Scale for Pain (NRS) 0-10. | Treatment participants = 100%  Placebo participants = 100% | 2 |  |  | Use pharmacological agent to treat muscle cramps. |
| 2 | Abd-Elsalam S. (2017)^48^ | NR. | NRS 0-10. | Treatment participants = 100%  Placebo participants = 100% | 2 |  |  | Use pharmacological agent to treat muscle cramps. |
| 3 | Elfert AA. (2016)^46^ | NR. | NRS 0-10. | Treatment participants = 100%  Placebo participants = 100% | 1 |  |  | Use pharmacological agent to treat muscle cramps. |
| 4 | Acharya C. (2017)^50^ | NR. | No validated pain assessment tool reported. | NR. | 4 |  |  | Use multimodal approach. |
| 5 | Acharya C. (1992)^51^ | NR. | No validated pain assessment tool reported. | NR. | 1 |  |  | Use pharmacological agent to treat ascites. |
| 6 | Afendy A. (2009)^52^ | NR. | No validated pain assessment tool reported. | NR. | 1 | 2 |  | No pain related recommendations reported. |
| 7 | Baumann AJ. (2015)^56^ | NR. | No validated pain assessment tool reported. | NR. | 2 | 2 |  | Use early palliative care intervention for advanced liver disease. |
| 8 | Dan AA. (2008)^62^ | NR. | No validated pain assessment tool reported. | NR. | 1 |  |  | No pain related recommendations reported. |
| 9 | Dan AA. (2006)^63^ | NR. | No validated pain assessment tool reported. | NR. | 1 | 2 |  | No pain related recommendations reported. |
| 10 | Macdonald S. (2019)^74^ | NR. | No validated pain assessment tool reported. | NR. | 1 |  |  | No pain related recommendations reported. |
| 11 | Randall HB. (2017)^80^ | NR. | No validated pain assessment tool reported. | NR. | 1 |  |  | Use multimodal approach. |
| 12 | Rogal S. (2019)^83^ | NR. | No validated pain assessment tool reported. | NR. | 1 |  |  | Use opioid sparring analgesic. |
| 13 | Rogal S. (2013)^82^ | Chronic pain.  Pain identified in multiple bodily regions. | NRS 0-10. | Total participants = 34% | 1 | 3 |  | Use targeted interventions that focus on modifiable factors. |
| 14 | Roth K. (2000)^87^ | NR. | Modified Likert scale  “How much of the time do you experience pain?” Responses were (1) not at all severe, (2) moderately severe, and (3) extremely severe. | Total participants = 30-40% | 1 | 2 |  | Use of targeted interventions that focus on pain. |
| 15 | Abd El-Wahab EW. (2016)^45^ | NR. | No validated pain assessment tool reported. | NR. |  | 1 |  | No pain related recommendations reported. |
| 16 | Angeli P. (1996)^53^ | NR. | No validated pain assessment tool reported. | NR. | 2 |  |  | Use pharmacological agent to treat cramps. |
| 17 | Barboza K. (2016)^54^ | NR. | No validated pain assessment tool reported. | NR. | 1 | 2 |  | Frequent assessments of physical, cognitive, and affective functioning. |
| 18 | Baskol M. (2014)^55^ | NR. | No validated pain assessment tool reported. | NR. | 2 |  |  | No pain related recommendations reported. |
| 19 | Bianchi G. (2005)^57^ | NR. | No validated pain assessment tool reported. | NR. | 3 | 2 |  | Use pharmacological agents to treat psychological symptoms. |
| 20 | Bondini S. (2007)^60^ | NR. | No validated pain assessment tool reported. | NR. | 1 | 2 |  | No pain related recommendations reported. |
| 21 | Chatrath H. (2012)^61^ | NR. | No validated pain assessment tool reported. | NR. | 1 | 1 |  | No pain related recommendations reported. |
| 22 | Evon D. (2016)^64^ | NR. | No validated pain assessment tool reported. | NR. |  | 1 |  | More detailed assessments for fatigue. |
| 23 | Fontana RJ. (2001)^65^ | NR. | No validated pain assessment tool reported. | NR. | 3 | 2 |  | No pain related recommendations reported. |
| 24 | Fritz E. (2009)^66^ | NR. | No validated pain assessment tool reported. | NR. | 1 | 2 |  | Treat psychological illness to control gastrointestinal symptoms. |
| 25 | Gallegos-Orozco JF. (2003)^67^ | NR. | No validated pain assessment tool reported. | NR. | 1 | 1 |  | Use education prior to treatments. |
| 26 | Gutteling JJ. (2007)^68^ | NR. | No validated pain assessment tool reported. | NR. | 1 | 2 |  | Use psychological interventions. |
| 27 | Gutteling JJ. (2006)^69^ | NR. | No validated pain assessment tool reported. | NR. | 2 | 2 |  | Use targeted interventions that focus on modifiable factors. |
| 28 | Hauser W. (2004)^71^ | NR. | No validated pain assessment tool reported. | NR. |  | 2 |  | Use valid assessment measures to evaluate side effects of pharmacological treatments. |
| 29 | Kallman J. (2007)^72^ | NR. | No validated pain assessment tool reported. | NR. |  | 1 |  | No pain related recommendations reported. |
| 30 | Kaltsakas G. (2013)^73^ | NR. | No validated pain assessment tool reported. | NR. | 1 |  |  | No pain related recommendations reported. |
| 31 | Madan A. (2012)^75^ | Chronic pain.  Pain identified in multiple bodily regions. | Brief Pain Inventory (BPI). | Total participants = 100%  (33% report pain > 2 body locations) | 4 |  |  | Use multimodal approach. |
| 32 | Marchesini G. (2001)^76^ | NR. | No validated pain assessment tool reported. | NR. | 4 |  |  | Reduce pharmacological agents.  More detailed assessments of cramps and pruritus. |
| 33 | Paglione HB. (2019)^77^ | NR. | No validated pain assessment tool reported. | NR. |  | 3 |  | Incorporate patient’s beliefs in coping strategies. |
| 34 | Perez-San-Gregorio MA. (2012)^78^ | NR. | No validated pain assessment tool reported. | NR. | 1 | 2 |  | Use psychological intervention. |
| 35 | Poonja Z. (2014)^79^ | NR. | No validated pain assessment tool reported. | Total participants = 65% | 1 | 2 |  | Use palliative care interventions. |
| 36 | Rodrigue JR. (2010)^81^ | NR. | No validated pain assessment tool reported. | NR. | 2 | 1 |  | No pain related recommendations reported. |
| 37 | Rogal S. (2015)^84^ | Chronic pain.  Pain identified in multiple bodily regions. | McGill Pain Questionnaire (MPQ). | Total participants = 79% | 2 | 2 |  | Research needed to systematically investigate pain interventions. |
| 38 | Rogal S. (2015)^85^ | NR. | McGill Pain Questionnaire (MPQ). | NR. | 2 | 2 |  | Use multimodal approach. |
| 39 | Rogal S. (2013)^86^ | NR. | NRS 0-10. | Hospitalized participants = 33%  Non-hospitalized participants = 18% | 2 | 2 |  | More detailed assessment of psychological factors of pain.  Reduce opioid use. |
| **Quantitative design: Descriptive studies** | | | | | | | | |
| 40 | Hansen L. (2014)^70^ | Chronic pain.  Pain identified in multiple bodily regions. | Brief Pain Inventory (BPI). | Total participants = 100% | 2 |  |  | More detailed assessments of pain characteristics, medications, and complimentary treatments. |
| **Mixed Methods Design** | | | | | | | | |
| 41 | Blackburn P. (2007)^58^ | NR. | No validated pain assessment tool reported. | NR. |  | 4 |  | Research needed to explore psychological interventions. |
| 42 | Blasiole JA. (2006)^59^ | NR. | No validated pain assessment tool reported. | NR. |  | 2 | 1 | Use educational support. |
| **Qualitative Design** | | | | | | | | |
| 43 | Abdi F. (2015)^49^ | NR. | No validated pain assessment tool reported. | NR. |  | 2 | 1 | Use educational, emotional, and social support. |
