# Supplementary material for: A scoping review to identify and map the multidimensional domains of pain in adults with advanced liver disease
Source: Can J Pain. 2020 Sep 15;4(1):210–24. doi: 10.1080/24740527.2020.1785855 (PMC7951148; doi:10.1080/24740527.2020.1785855)
Supplement: Supplemental Material [file UCJP_A_1785855_SM7867.docx]

Supplemental Appendix D. Quality appraisal of included studies using Mixed Methods Appraisal Tool (MMAT) version 2018^43^

SUMMARY RESULTS (see Supplemental Appendix E for individual study results)

|  | Yes | | No | | Can’t  tell | |
| --- | --- | --- | --- | --- | --- | --- |
|  | n | % | n | % | n | % |
|  |  |  |  |  |  |  |
| *Generic questions for all studies*^45-87^ |  |  |  |  |  |  |
| S1. Are there clear research questions? | 6 | 14% | 37 | 86% | 0 | 0% |
| S2. Do the collected data allow to address the research questions? | 43 | 100% | 0 | 0% | 0 | 0% |
|  |  |  |  |  |  |  |
| *Qualitative design*^49^ |  |  |  |  |  |  |
| 1.1 Is the qualitative approach appropriate to answer the research question? | 1 | 100% | 0 | 0% | 0 | 0% |
| 1.2 Are the qualitative data collection methods adequate to address the research questions? | 0 | 100% | 1 | 100% | 0 | 0% |
| 1.3 Are the findings adequately derived from the data? | 1 | 100% | 0 | 0% | 0 | 0% |
| 1.4 Is the interpretation of results sufficiently substantiated by data? | 0 | 0% | 1 | 100% | 0 | 0% |
| 1.5 Is there coherence between qualitative data sources, collection, analysis, and interpretation? | 1 | 100% | 0 | 0% | 0 | 0% |
|  |  |  |  |  |  |  |
| *Quantitative design: randomized controlled trials*^46-48^ |  |  |  |  |  |  |
| 2.1 Is randomization appropriately performed? | 3 | 100% | 0 | 0% | 0 | 0% |
| 2.2 Are the groups comparable at baseline? | 0 | 0% | 3 | 100% | 0 | 0% |
| 2.3 Are there complete outcome data? | 2 | 67% | 1 | 33% | 0 | 0% |
| 2.4 Are outcome assessors blinded to the intervention provided? | 3 | 100% | 0 | 0% | 0 | 0% |
| 2.5 Did the participants adhere to the assigned intervention? | 3 | 100% | 0 | 0% | 0 | 0% |
|  |  |  |  |  |  |  |
| *Quantitative design: non-randomized studies*^45,50-57,60-69,71-87^ |  |  |  |  |  |  |
| 3.1 Are the participants representative of the target population? | 8 | 22% | 28 | 78% | 0 | 0% |
| 3.2 Are measurements appropriate regarding both the outcome and exposure/intervention? | 34 | 95% | 2 | 5% | 0 | 0% |
| 3.3 Are there complete outcome data? | 35 | 97% | 1 | 3% | 0 | 0% |
| 3.4 Are the confounders accounted for in the design analysis? | 19 | 53% | 16 | 45% | 1 | 2% |
| 3.5 During the study period, is the intervention/exposure administered as intended? | 34 | 94% | 2 | 6% | 0 | 0% |
|  |  |  |  |  |  |  |
| *Quantitative design: descriptive studies*^70^ |  |  |  |  |  |  |
| 4.1 Is the sampling strategy relevant to address the research question? | 1 | 100% | 0 | 0% | 0 | 0% |
| 4.2 Is the sample representative of the target population? | 0 | 0% | 1 | 100% | 0 | 0% |
| 4.3 Are the measurements appropriate? | 1 | 100% | 0 | 0% | 0 | 0% |
| 4.4 Is the risk of nonresponse bias low? | 1 | 100% | 0 | 0% | 0 | 0% |
| 4.5 Is the statistical analysis appropriate to answer the research question? | 1 | 100% | 0 | 0% | 0 | 0% |
|  |  |  |  |  |  |  |
| *Mixed Methods*^58,59^ |  |  |  |  |  |  |
| 5.1 Is there an adequate rationale for using a mixed methods design to address the research question? | 0 | 0% | 2 | 100% | 0 | 0% |
| 5.2 Are the different components of the study effectively integrated to answer the research question? | 2 | 100% | 0 | 0% | 0 | 0% |
| 5.3 Are the results adequately brought together into overall interpretations? | 2 | 100% | 0 | 0% | 0 | 0% |
| 5.4 Are divergences and inconsistencies between quantitative and qualitative results adequately addressed? | 2 | 100% | 0 | 0% | 0 | 0% |
| 5.5 Do the different components of the study adhere to the quality criteria of each tradition of the methods involved? | 0 | 0% | 2 | 100% | 0 | 0% |
