# Supplementary material for: A scoping review to identify and map the multidimensional domains of pain in adults with advanced liver disease
Source: Can J Pain. 2020 Sep 15;4(1):210–24. doi: 10.1080/24740527.2020.1785855 (PMC7951148; doi:10.1080/24740527.2020.1785855)
Supplement: Supplemental Material [file UCJP_A_1785855_SM7862.docx]

Supplemental Appendix E. Quality appraisal of included studies using Mixed Methods Appraisal Tool (MMAT) version 2018^43^

INDIVIDUAL STUDY RESULTS (see Supplemental Appendix D for summary results)

|  | First Author, Year | S1. Are there clear research questions? | S2. Do the collected data allow to address the research questions? | 2.1 Is randomization appropriately performed? | 2.2 Are the groups comparable at baseline? | 2.3 Are there complete outcome data? | 2.4 Are outcome assessors blinded to the intervention provided? | 2.5 Did the participants adhere to the assigned intervention? | Comments | MMAT^43^ Quality appraisal |
| --- | --- | --- | --- | --- | --- | --- | --- | --- | --- | --- |
| **Quantitative design: randomized controlled trials** | | | | | | | | | | |
| 1 | Abd-Elsalam S. (2018)^47^ | No | Yes | Yes | No | Yes | Yes | Yes | S1 Research questions not reported. Purpose of study reported. "The aim of this study was to evaluate the efficacy and safety of methocarbamol as a novel therapy in controlling muscle cramps in cirrhotic patients."^47(p499)^  2.2 Single site. Some concerns with representativeness of sample. "This study was carried out in a major University Hospital in Egypt..."^47(p499)^  2.2 Severity liver disease not reported.  "Patients with liver cirrhosis in addition to chronic hepatitis C complaining of frequent muscle cramps for at least 1 month were included in the study."^47(p500)^ | Threats to external validity including single center design and sample representativeness.  Risk for confounding bias due to lack of control for disease severity. |
| 2 | Abd-Elsalam S. (2017)^48^ | No | Yes | Yes | No | Yes | Yes | Yes | S1 Research questions not reported. Purpose of study reported. "The aim of the study was to assess the efficacy and safety of orphenadrine in the treatment of muscle cramps in patients with liver cirrhosis."^48(p423)^  2.2 Single site. Some concerns with representativeness of sample. "The patients had been admitted at the Department of Tropical Medicine and Infectious diseases in Tanta University Hospital."^48(p423)^  2.2 Severity liver disease not reported.  "Inclusion criteria included patients with liver cirrhosis and frequent muscle cramps."^48(p423)^ | Threats to external validity including single center design and sample representativeness.  Risk for confounding bias due to lack of control for disease severity. |
| 3 | Elfert AA. (2016)^46^ | No | Yes | Yes | No | No | Yes | Yes | S1 Research questions not reported. Purpose of study reported. "...to assess the safety and effectiveness of baclofen in the treatment of muscle cramps in patients with liver cirrhosis."^46(p1280)^  2.2 Single site. Some concerns with representativeness of sample. "...A total of 127 cirrhotic patients with muscle cramps were enrolled in this study who had been admitted at the Department of Tropical Medicine and Infectious diseases in Tanta University Hospital."^46(p1281)^  2.3 Missing data not reported. "A total of 127 cirrhotic patients with muscle cramps were enrolled...Of these, 100 patients...had been assigned."^46(p1281)^ | Threats to external validity including single center design and sample representativeness.  Incomplete outcome data. |

| **Quantitative design: non-randomized studies** | | | | | | | | | |  |
| --- | --- | --- | --- | --- | --- | --- | --- | --- | --- | --- |
|  | First Author, Year | S1. Are there clear research questions? | S2. Do the collected data allow to address the research questions? | 3.1 Are the participants representative of the target population? | 3.2 Are measurements appropriate regarding both the outcome and exposure/intervention? | 3.3 Are there complete outcome data? | 3.4 Are the confounders accounted for in the design analysis? | 3.5 During the study period, is the intervention/exposure administered as intended? | Comments | MMAT^34^ Quality appraisal |
| 4 | Acharya C. (2017)^50^ | No | Yes | No | Yes | Yes | Yes | Yes | S1 Research questions not reported. Hypothesis provided. "We hypothesized that opioid use on hospital discharge is associated with a higher risk of readmissions…"^50(p320)^  3.1 Single site cohort. Some concerns with representativeness of sample. "...cohort 1 consisted of cirrhotic patients who were hospitalized for non-elective reasons at VCU Medical Center."^50(p320)^ | Risk of bias including single center design and unclear sample representativeness. |
| 5 | Acharya C. (1992)^51^ | No | Yes | No | Yes | Yes | No | No | S1 Research questions not reported. Purpose of study reported. "Thus, we evaluated the efficacy of a low-cost, easily available synthetic colloid-like, low molecular weight dextran infusion as the plasma expander."^51(p31)^  3.1 Single site. Some concerns with representativeness of sample. "...sixty-five patients with liver cirrhosis and tense ascites were admitted to the Rajgarhia Liver unit."^51(p31)^ "Of the 65 cirrhotics with tense ascites evaluated, 40...were included in the study."^51(p32)^  3.4 The procedures for assessors blinded to intervention not discussed. "Patients with and without pedal edema were separately randomized to receive either therapeutic schedule A or B."^51(p32)^  3.5 There is a lack of discussion commenting on the consistency in the delivery of the planned intervention for all the participants.  "During the trial, all patients were fed on a 2,000 calorie/day diet, containing 60g of vegetable protein, and 2 g of salt."^51(p32)^ | Risk of bias including single center design and unclear sample representativeness.  The procedures for assessors blinded to intervention not discussed.  There is a lack of discussion commenting on the consistency in the delivery of the planned intervention for all the participants. |
| 6 | Afendy A. (2009)^52^ | No | Yes | Yes | Yes | Yes | Yes | Yes | S1 Research questions not reported. Purpose of study reported. "This study aims to explore the impact of different types of liver disease and clinicodemographic factors on HRQL in a large cohort of patients with CLD."^52(p470)^ | See supplemental online material A and B. |
| 7 | Baumann AJ. (2015)^56^ | No | Yes | No | Yes | No | Yes | No | S1 Research questions not reported. Purpose of study reported. "...we implemented a longitudinal, multidisciplinary, and early palliative care intervention with ESLD patients awaiting liver transplant."^56(p883)^  3.1 Single site. Some concerns with the representativeness of the sample.  "...EPCI was incorporated into the standard one-week pretransplant evaluation process at the Liver Transplantation Center of Albert Einstein Medical Center in Philadelphia."^56(p883)^ "The remaining 50 (63.3%) patients completed the transplant evaluation including EPCI."^56(p883)^  3.3 Limited follow up data outcome data. "The remaining 50 (63.3%) patients completed the transplant evaluation including EPCI."^56(p883)^  3.5 There is a lack of discussion commenting on the consistency in the delivery of the planned intervention for all the participants.  "The palliative care encounter followed the hepatology visit and allowed the patient to address fears and concerns about their hepatology treatment plan."^56(p883)^ | Risk of bias including single center design and unclear sample representativeness.  Incomplete outcome data.  There is a lack of discussion commenting on the consistency in the delivery of the planned intervention for all the participants. |
| 8 | Dan AA. (2008)^62^ | No | Yes | Yes | No | Yes | Yes | Yes | S1 Research questions not reported. Purpose of study reported. "...we assess the health utilities of patients with chronic liver disease...We also compare utility scores among patients with different types of liver disease."^62(p322)^  3.2 There is a lack of discussion outlining the participants' eligibility criteria. "One hundred forty patients were identified from the database that had HRQL data available..."^62(p322)^ | Risk of bias with unclear sample representativeness. There is a lack of discussion outlining the participants' eligibility criteria. |
| 9 | Dan AA. (2006)^63^ | No | Yes | No | Yes | Yes | Yes | Yes | S1 Research questions not reported. Purpose of study reported. "...the association of HRQL with depressive symptoms, and anemia were assessed."^63(p492)^  3.1 There is a lack of discussion outlining all the participants eligible. "Two hundred and seventy-one patients with CH-C who received a regimen of pegylated interferon alfa 2b and ribavirin were included in this study."^63(p492)^ | Risk of bias with unclear sample representativeness. There is a lack of discussion outlining the participants’ eligibility criteria. |
| 10 | Macdonald S. (2019)^74^ | No | Yes | Yes | Yes | Yes | Yes | Yes | S1 Research questions not reported. Purpose of study reported. "The aims of this study were to determine the association between HRQL, using the SF-36, and mortality in patients with severe ascites."^74(p322)^ | See supplemental online material A and B. |
| 11 | Randall HB. (2017)^80^ | No | Yes | No | Yes | Yes | Yes | Yes | S1 Research questions not reported. Purpose of study reported. "Our goals were to quantify fills for prescription opioids on the waiting list, identify correlates of opioid use, and determine whether prescription opioid exposure before and after transplant is associated with posttransplant outcomes."^80(p306)^  3.1 Some concerns with representativeness of the target population. Severity of liver disease not reported.  "LT recipients who were eligible for the study had SRTR records of LT...had available pharmaceutical fill records while on the transplant waiting list."^80(p307)^ | Risk of bias with unclear sample representativeness. Severity of liver disease not reported. |
| 12 | Rogal S. (2019)^83^ | No | Yes | Yes | Yes | Yes | Yes | Yes | S1 Research questions not reported. Purpose of study reported. "The objective of this study was to assess the longitudinal trends and predictors of opioid prescribing in a large national sample of veterans with cirrhosis."^83(p1166)^ | See supplemental online material A and B. |
| 13 | Rogal S. (2013)^82^ | No | Yes | Yes | Yes | Yes | Can’t tell | Yes | S1 Research questions not reported. Purpose of study reported. "...the purpose of this investigation was to understand factors associated with pain and its treatment in patients with chronic liver disease."^82(p2977)^  3.4 Unable to tell confounders in sampling bias. "A consecutive sample of outpatients seen in the Center for Liver Diseases over a 2-month period."^82(p2977)^ | Risk of bias with unclear sample representativeness. |
| 14 | Roth K. (2000)^87^ | No | Yes | No | Yes | Yes | Yes | Yes | S1 Research questions not reported. Purpose of study reported. "To understand patterns of care and end-of-life preferences for patients dying with end stage liver disease with cirrhosis."^87(pS122)^  3.1 Severity of liver disease not reported. "Inclusion criteria for ESLDC consistent of chart documentation of cirrhosis and at least two of the following: a serum albumin level of 3.0 mg/dL or less, uncontrolled ascites, hepatic encephalopathy, documentation of cachexia, or a massive upper gastrointestinal bleed."^87(pS123)^ | Risk for confounding bias due to lack of control for disease severity. |
| 15 | Abd El-Wahab EW. (2016)^45^ | No | Yes | No | Yes | Yes | Yes | Yes | S1 Research questions not reported. Purpose of study reported. "…the impact of chronic viral hepatitis on the HRQOL of Egyptian patients compared it with the impact of the same on the HRQOL of an interventional group of patients undergoing IFN therapy and a control group of noninfected individuals using HRQOL specific assessment instruments focusing on liver disease."^45(p153)^  3.1 Severity of liver disease not reported. "The criteria for inclusion...liver biopsy showing Ishak stage from 1 to 4."^45(p153)^ | Risk for confounding bias due to lack of control for disease severity. |
| 16 | Angeli P. (1996)^53^ | No | Yes | No | Yes | Yes | No | Yes | S1 Research questions not reported. Purpose of study reported. "The aims of this study were to evaluate the prevalence of cramps in cirrhosis according to cause and severity of the disease."^53(p264)^  3.1 Some concerns with the representativeness of the sample. "...consecutive cirrhotic patients admitted to our Institute were examined for the study."^53(p264)^  3.4 Unable to tell confounders in sampling bias. There is a lack of clear discussion of the process of obtaining the participants.. "224 consecutive cirrhotic patients admitted...for the study...One hundred seventy-one controls and 171 cirrhotic patients were included in the study."^53(p264,266)^ | Risk of bias with unclear sample representativeness.  Unclear confounders in sampling bias. There is a lack of clear discussion of the process of obtaining the participants. |
| 17 | Barboza K. (2016)^54^ | No | Yes | No | Yes | Yes | Yes | Yes | S1 Research questions not reported. Purpose of study reported. "...we assess the relationship between depression, HE, and HRQOL among cirrhotic patients with HCV."^54(p870)^  3.1 Lack of discussion outlining all eligible participants (e.g., how many did not participate).^54(p870-871)^  3.1 Some concerns with the representativeness of the sample. "Forty-three ambulatory patients with cirrhosis."^54(p870)^ | Lack of discussion outlining all eligible participants. |
| 18 | Baskol M. (2014)^55^ | No | Yes | No | Yes | Yes | No | Yes | S1 Research questions not reported. Purpose of study reported. "...to assess the frequency of true muscle cramps in non-alcoholic cirrhotic patients as well as the correlation of plasma zinc levels with muscle cramps in patients with cirrhosis."^55(p524)^  3.1 Single site. Some concerns with representativeness of the sample. "This prospective study was performed on 100 cirrhotic patients admitted to the gastroenterology clinic of Erciyes University Hospital."^55(p524)^  3.4 Some concerns with confounders of nonresponse bias. There is a lack of discussion outlining all the participants eligible (e.g., how many excluded).^55(p524-525)^ | Risk of bias with single center design Some concerns with representativeness of the sample.  Some concerns with confounders of nonresponse bias. There is a lack of discussion outlining all the participants eligible. |
| 19 | Bianchi G. (2005)^57^ | No | Yes | No | Yes | Yes | No | Yes | S1 Research questions not reported. Purpose of study reported. "Our study was aimed at measuring psychological well-being in patients with liver cirrhosis."^57(p594)^  3.1 Some concerns with the representativeness of the sample. "One hundred and fifty-six consecutive patients with liver cirrhosis."^57(p594)^  3.4 Some concerns with the confounding nonresponse bias. There is a lack of discussion concerning the 156 enrolled participants with only 150 completing the BDI questionnaire.^57(p594,596)^ | Risk of bias with unclear sample representativeness.  Some concerns with the confounding nonresponse bias. There is a lack of discussion concerning nonresponse participants. |
| 20 | Bondini S. (2007)^60^ | No | Yes | Yes | Yes | Yes | No | Yes | S1 Research questions not reported. Purpose of study reported. "Compare HRQL between patients with chronic hepatitis B, chronic hepatitis C, primary biliary cirrhosis, and healthy controls."^60(p1119)^  3.4 Some concerns with the confounding nonresponse bias. There is a lack of discussion outlining the eligibility of all participants (e.g., how many excluded).^60(p1120)^ | Risk of bias with confounding nonresponse bias. There is a lack of discussion outlining the eligibility of all participants. |
| 21 | Chatrath H. (2012)^61^ | Yes | Yes | No | Yes | Yes | Yes | Yes | 3.1 Severity of liver disease not reported. "Patients with an established diagnosis of cirrhosis (clinical, histologic, or radiologic) as determined by the treating hepatologist…"^61(p2)^  3.1 Some concerns with representativeness of the sample. There is a lack of discussion concerning the sample strategy.^61(p2,3)^  3.1 Some concerns with representativeness of the sample. "A total of 150 adult patients with cirrhosis were enrolled consecutively."^61(p1)^ | Risk of bias with unclear sample representativeness.  There is a lack of discussion concerning the sample strategy. |
| 22 | Evon D. (2016)^64^ | Yes | Yes | No | Yes | Yes | Yes | Yes | 3.1 Some concerns with representativeness of the sample. "The HBRN observational cohort study enrolls participants with hepatitis B.”^64(p1087)^  3.1 Severity of liver disease not reported. | Risk of bias with unclear sample representativeness.  Risk for confounding bias due to lack of control for disease severity. |
| 23 | Fontana RJ. (2001)^65^ | No | Yes | Yes | Yes | Yes | No | Yes | S1 Research questions not reported. Purpose of study reported. "The aim of this study was to determine whether variability in HRQOL scores in a cohort of patients with compensated chronic hepatitis C who had failed previous interferon therapy could be explained in part by demographic and extrahepatic clinical variables."^65(p170)^  3.4 Some concerns with confounding nonresponse bias. There is a lack of discussion outlining the participants eligible for the study (e.g., how many did not participate).^65(p171)^ | Risk of confounding non-response bias. There is a lack of discussion outlining the participants eligible for the study. |
| 24 | Fritz E. (2009)^66^ | No | Yes | No | Yes | Yes | No | Yes | S1 Research questions not reported. Purpose of study reported. "...to evaluate the prevalence of Gi symptoms with liver cirrhosis and to determine whether GI symptoms in liver cirrhotic patients can affect QoL and psychological well being in liver cirrhosis."^66(p371)^  3.1 Some concerns with the representativeness of the sample. "...consecutive patients with liver cirrhosis were asked."^66(p371)^  3.4 Some concerns with the confounding nonresponse bias. There is a lack of discussion outlining the eligibility of participants (e.g., how many refused).^66(p371)^ | Risk of bias with unclear sample representativeness.  Some concerns with the confounding nonresponse bias. There is a lack of discussion outlining the eligibility of participants. |
| 25 | Gallegos-Orozco JF. (2003)^67^ | No | Yes | No | Yes | Yes | No | Yes | S1 Research questions not reported. Purpose of study reported. "The aim of the present study was to evaluate HRQL and depression in a sample of Mexican chronic hepatitis C patients."^67(p125)^  3.1 Some concerns with the representativeness of the sample. There is a lack of discussion on the sampling strategy.^67(p125)^  3.4 Some concerns with confounding nonresponse bias. There is a lack of discussion outlining the participants eligible for the study (e.g., how many refused).^67(p125)^ | Risk of bias with unclear sample representativeness. There is a lack of discussion on the sampling strategy.  Some concerns with confounding nonresponse bias. There is a lack of discussion outlining the participants eligible for the study. |
| 26 | Gutteling JJ. (2007)^68^ | No | Yes | No | Yes | Yes | No | Yes | S1 Research questions not reported. Purpose of study reported. "…assess the relative levels of HRQoL of patients awaiting liver transplantation…assess the relationships between HRQoL and several psychological variables such as anxiety, depression, and coping in liver transplant candidates."^68(p438)^  3.1 Some concerns with the representativeness of the sample. "In total, 44 patients participated."^68(p439)^  3.4 Some concerns with the confounding nonresponse bias. "Out of the 61 patients...44 participated."^68(p439)^ | Threats to bias with unclear sample representativeness.  Some concerns with the confounding nonresponse bias. |
| 27 | Gutteling JJ. (2006)^69^ | No | Yes | No | Yes | Yes | No | Yes | S1 Research questions not reported. Purpose of study reported. "...assess the impact of physical and psychosocial determinant on a weighted score of health-related quality of life in patients with chronic liver disease."^69(p1629)^  3.1 Some concerns with the representativeness of the sample. There is a lack of discussion outlining the eligibility criteria.^69(p1631)^  3.4 Some concerns with confounding nonresponse bias. "A total of 2020 members of the NLV were approached...1175 respondents were included in the study."^69(p1631)^ | Risk of bias with unclear sample representativeness. There is a lack of discussion outlining the eligibility criteria.  Some concerns with confounding nonresponse bias. |
| 28 | Hauser W. (2004)^71^ | No | Yes | No | Yes | Yes | No | Yes | S1 Research questions not reported. Purpose of study reported. "The study aims to assess influence of the liver disease, active medical and psychiatric comorbidities, and sociodemographic variables in the determination of health-related quality of life."^71(p157)^  3.1 Some concerns with the representativeness of the sample. "...consecutive patients approached..."^71(p159)^  3.4 Some concerns with confounding nonresponse bias. There is incomplete information provided where the study suggests 255 participants approached, and 203 patients agreed to participate. It accounts for 24 patients excluded for exclusionary reasons, however, does not account for the 28 participants who were excluded.^71(p159)^ | Risk of bias with unclear sample representativeness.  Some concerns with confounding nonresponse bias. There is incomplete information provided. |
| 29 | Kallman J. (2007)^72^ | No | Yes | No | Yes | Yes | Yes | Yes | S1 Research questions not reported. Purpose of study reported. "…evaluate the potential impact of a variety of clinicodemographic variables on patients' well-being."^72(p2532)^  3.1 Severity of liver disease not reported. "Diagnosis of chronic hepatitis C was based on the presence of hepatitis C antibody, elevated ALT and detectable HCV RNA."^72(p2532)^  3.1 Single site. Some concerns with the representativeness of the sample. "Consecutive patients with the diagnosis of chronic hepatitis C."^72(p2532)^ | Risk of bias including single center design and unclear sample representativeness. |
| 30 | Kaltsakas G. (2013)^73^ | No | Yes | No | Yes | Yes | Yes | Yes | S1 Research questions not reported. Purpose of study reported. "...to determine the prevalence of chronic dyspnea and the interrelationships among chronic dyspnea, measured with the modified Medical Research Council scale, respiratory muscle strength, and lung function in patients with end-stage liver disease."^73(p57)^  3.1 Some concerns with the representativeness of the sample.  "Forty of them were included in this preliminary study."^64(p57)^ "Sixty-eight consecutive, ambulatory, Caucasian patients."^73(p57)^ | Risk of bias with unclear sample representativeness. |
| 31 | Madan A. (2012)^75^ | Yes | Yes | No | Yes | Yes | Yes | Yes | 3.1 Single site. Some concerns with the representativeness of the sample.  "The study sample consisted of 108 adult, outpatient liver transplant candidates at a large academic medical center in the southern United States."^75(p380)^ | Risk of bias including single center design and unclear sample representativeness. |
| 32 | Marchesini G. (2001)^76^ | No | Yes | Yes | Yes | Yes | Yes | Yes | S1 Research questions not reported. Purpose of study reported. "We administered these questionnaires to a well-characterized sample of patients with cirrhosis."^76(p171)^ | See supplemental online material A and B. |
| 33 | Paglione HB. (2019)^77^ | No | Yes | No | No | Yes | No | Yes | S1 Research questions not reported. Purpose of study reported. "To evaluate quality of life, religiosity and anxiety and depressive symptoms in liver transplant candidates."^77(p1)^  3.1 Some concerns with the representativeness of the sample.  "The sample was by convenience."^77(p2)^ "Thus, the total sample consisted of 50 patients."^77(p2)^  3.2 There is a lack of discussion outlining the rationale for selecting the instruments used in the study.^77(p2-3)^  3.4 Some concerns with confounders nonresponse bias. The 50 (74%) participants from the 67 eligible may not be a representative of the possible 1235 enrolled in the liver transplant list.^77(p2)^ | Risk of bias with unclear sample representativeness.  There is a lack of discussion outlining the rationale for selecting the instruments used in the study.  Some concerns with confounding nonresponse bias. Participants may not be representative of the target population. |
| 34 | Perez-San-Gregorio MA. (2012)^78^ | No | Yes | No | Yes | Yes | No | Yes | S1 Research questions not reported. Purpose of study reported. "To compare the biopsychosocial functioning among liver transplantation and cirrhotic patients as a function of self-perceived pain level."^78(p2612)^  3.1 Some concerns with the representativeness of the sample. Sampling strategy not reported.^78(p2613)^  3.4 Some concerns with confounding nonresponse bias. Participant response rate not discussed (e.g., reason for refusal).^78(p2613)^ | Risk of bias with unclear sample representativeness. Sampling strategy not reported.  Some concerns with confounding nonresponse bias. Participant response rate not discussed. |
| 35 | Poonja Z. (2014)^79^ | Yes | Yes | No | Yes | Yes | No | Yes | 3.1 Single site. Some concerns with the representativeness of the sample. "This single-center retrospective cohort study..."^79(p695)^  3.4 Some concerns with confounders with nonresponse bias. "...302 patients were identified...Complete data were found on 102 patients…One hundred ninety-five patients were excluded because there were followed by other centers…Five patients were subsequently excluded; 4 underwent transplantation at other centers, and 1 patient recovered from acute liver failure."^79(p694)^ | Risk of bias including single center design and unclear sample representativeness.  Some concerns with confounders with nonresponse bias. |
| 36 | Rodrigue JR. (2010)^81^ | Yes | Yes | No | Yes | Yes | Yes | Yes | 3.1 Some concerns with the representativeness of the sample.  "Using a convenience sampling strategy."^81(p222)^ | Risk of bias with unclear sample representativeness. |
| 37 | Rogal S. (2015)^84^ | Yes | Yes | No | Yes | Yes | Yes | Yes | 3.1 Single site. Some concerns with the representativeness of the sample. "We screened the medical records of patients scheduled for outpatient hepatology appointments."^84(p1010)^ | Risk of bias including single site design and unclear sample representativeness. |
| 38 | Rogal S. (2015)^85^ | No | Yes | No | Yes | Yes | No | Yes | S1 Research questions not reported. Purpose of study reported. "...the aims of this investigation were to: (1) determine the prevalence of fibromyalgia using a validated diagnostic instrument in patients with three different etiologies for liver cirrhosis; and (2) investigate clinical correlates of fibromyalgia in the total sample."^85(p1483)^  3.1 Single site. Some concerns with the representativeness of the sample.  "A total of 1,551 patients were scheduled to be seen at the Center for Liver Diseases over the 3-month period of recruitment."^85(p1484)^  3.4 Some concerns with confounders of nonresponse bias. There is a lack of discussion specifying the 326 possible participants and 210 were recruited (e.g., what happened to 116 participants).  "Of the 326 possible subjects, 210 participants were recruited, 193 people making up the final cohort after 17 were excluded after consent because they were found later to meet exclusion criteria."^85(p1484)^ | Risk of bias including single center design and unclear sample representativeness.  Some concerns with confounders of nonresponse bias. There is a lack of discussion specifying nonresponse sample. |
| 39 | Rogal S. (2013)^86^ | No | Yes | No | Yes | Yes | No | Yes | S1 Research questions not reported. Purpose of study reported. "The aim of this study was to assess factors associated with healthcare utilization in patients with chronic liver disease with a focus on pain, opioid use and psychiatric symptoms."^86(p1497)^  3.1 Single site. Some concerns with the representativeness of the sample. "...retrospectively assessed a consecutive sample of 1286 visitors to a hepatology clinic."^86(p1497)^  3.4 Some concerns with confounders of nonresponse bias. There is a lack of discussion for excluding participants. Reasons given for 98 + 24 + 20 = 142 patients. Study had a total of 151 participants (9 participants not reported).^86(p1499)^ | Risk of bias including single site design and unclear sample representativeness.  Some concerns with confounders of nonresponse bias. There is a lack of discussion for excluding participants. |

| **Quantitative design: descriptive studies** | | | | | | | | | |  |
| --- | --- | --- | --- | --- | --- | --- | --- | --- | --- | --- |
|  | First Author, Year | S1. Are there clear research questions? | S2. Do the collected data allow to address the research questions? | 4.1 Is the sampling strategy relevant to address the research question? | 4.2 Is the sample representative of the target population? | 4.3 Are the measurements appropriate? | 4.4 Is the risk of nonresponse bias low? | 4.5 Is the statistical analysis appropriate to answer the research question? | Comments | MMAT^34^ Quality appraisal |
| 40 | Hansen L. (2014)^70^ | No | Yes | Yes | No | Yes | Yes | Yes | S1 Research questions not reported. Purpose of study reported. "The purpose of this study was to provide a longitudinal description of pain characteristics in outpatients with ESLD."^70(p33)^  4.2 Some concerns with the representativeness of the sample. "We included 20 hepatology clinic outpatients with ESLD in our sample."^70(p33)^ | Risk of bias with sample representativeness. |

|  | | | **Mixed methods design** | | | | | | | | |
| --- | --- | --- | --- | --- | --- | --- | --- | --- | --- | --- | --- |
|  | First Author, Year | S1. Are there clear research questions? | | S2. Do the collected data allow to address the research questions? | 5.1 Is there an adequate rationale for using a mixed methods design to address the research question? | 5.2 Are the different components of the study effectively integrated to answer the research question? | 5.3 Are the results adequately brought together into overall interpretations? | 5.4 Are divergences and inconsistencies between quantitative and qualitative results adequately addressed? | 5.5 Do the different components of the study adhere to the quality criteria of each tradition of the methods involved? | Comments | MMAT^34^ Quality appraisal |
| 41 | Blackburn P. (2007)^58^ | No | | Yes | No | Yes | Yes | Yes | No | S1 Research questions not reported. Purpose of study reported.  "The aims of this study were to explore psychological parameters in fatigued and non-fatigued PBC patients."^58(p654)^  5.1 Methods for analysis did not clearly incorporate qualitative and quantitative data. Rationale for mixed methods design not clearly explained. "From the semistructured interview, a perceived triggers scale was developed."^58(p655)&(p656-657)^  5.5 Lack of discussion on missing participant data. "The potential study participants were 30 consecutive patients attending a specialist PBC clinic who met the study criteria, 24 of whom were enrolled in the study."^58(p655)^ | Lack of methodological congruence. Methods for analysis did not clearly incorporate qualitative and quantitative data. Rationale for mixed methods design not clearly explained.  Lack of discussion on missing participant data. |
| 42 | Blasiole JA. (2006)^59^ | No | | Yes | No | Yes | Yes | Yes | No | S1 Research questions not reported. Purpose of study reported.  "...to characterize the nature and the causes of social problems as an important first step in alerting healthcare providers of the major stressors these patients face."^59(p4666)^  5.1 Rationale for mixed methods design not clearly explained. "Using mixed quantitative and qualitative methods, we propose to characterize the nature and the causes..."^59(p4666)^  5.5 Severity of liver disease not reported. "Patients with a confirmed diagnosis of HCV were invited to participate on the day of their clinic visit."^59(p4666)^  5.5 Some concerns with missing outcome data.  “A total of 499 patients were approached…Of the 403 patients successfully entered into the study…This left 342 patients in the total sample.”^59(p4667)^ | Rationale for mixed methods design not clearly explained.  Incomplete outcome data. |

| **Qualitative design** | | | | | | | | | |  |
| --- | --- | --- | --- | --- | --- | --- | --- | --- | --- | --- |
|  | First Author, Year | S1. Are there clear research questions? | S2. Do the collected data allow to address the research questions? | 1.1 Is the qualitative approach appropriate to answer the research question? | 1.2 Are the qualitative data collection methods adequate to address the research questions? | 1.3 Are the findings adequately derived from the data? | 1.4 Is the interpretation of results sufficiently substantiated by data? | 1.5 Is there coherence between qualitative data sources, collection, analysis, and interpretation? | Comments | MMAT^34^ Quality appraisal |
| 43 | Abdi F. (2015)^49^ | No | Yes | Yes | No | Yes | No | Yes | S1 Research questions not reported. Purpose of study reported. "The purpose of this study was to describe the experiences of individuals with liver cirrhosis."^49(p253)^  1.2 Convenience sampling. Some concerns with representativeness of sample. Sampling method may not be representative of other patients with liver cirrhosis. "A convenience sample was recruited of participants."^49(p253)^  1.4 There is limited participant quotes to support the results. | Lack of substantial data to support results.  Limited representatives of the sample with the utility of convenience sampling. |
